# Federated Metadata-Constrained iRadonMAP Framework with Mutual Learning for All-in-One Computed Tomography Imaging

**DOI:** 10.34133/cbsystems.0376

**Published:** 2025-08-27

**Authors:** Hao Wang, Xiaoyu Zhang, Hengtao Guo, Xuebin Ren, Shipeng Wang, Fenglei Fan, Jianhua Ma, Dong Zeng

**Affiliations:** ^1^ School of Biomedical Engineering, Southern Medical University, Guangzhou 510515, China.; ^2^ Seattle, WA, USA.; ^3^ National Engineering Laboratory for Big Data Analytics, Xi’an Jiaotong University, Xi’an 710049, China.; ^4^ School of Life Science and Technology, Xi’an Jiaotong University, Xi’an 710049, China.; ^5^ Department of Mathematics, The Chinese University of Hong Kong, Hong Kong, China.

## Abstract

With the increasing use of computed tomography (CT), concerns about radiation dose have grown. Deep-learning-based methods have shown great promise in improving low-dose CT image quality while further reducing patient dose. However, most deep-learning-based methods are trained on vendor-specific CT datasets with varying imaging conditions and dose levels, which results in poor generalizability across vendors due to marked data heterogeneity. Moreover, the centralization of multicenter datasets is restricted by the high costs of data collection and privacy regulations. To overcome these challenges, we propose FedM2CT, a federated metadata-constrained method with mutual learning for all-in-one CT reconstruction. This method enables simultaneous reconstruction of multivendor CT images with different imaging geometries and sampling protocols in one framework. Specifically, FedM2CT consists of 3 modules: task-specific iRadonMAP (TS-iRadonMAP), condition-prompted mutual learning (CPML), and federated metadata learning (FMDL). TS-iRadonMAP performs task-specific low-dose reconstruction, CPML shares condition-prompted knowledge between clients and the server, and FMDL aggregates model parameters with a metamodel to effectively mitigate the effect of data heterogeneity. Extensive experiments under 3 different settings demonstrate that the proposed FedM2CT achieves outstanding results compared to other methods, both qualitatively and quantitatively, showing the potential to achieve the goal of all-in-one CT reconstruction with different low-dose tasks, i.e., low-milliampere-second, sparse-view, and limited-angle.

## Introduction

Computed tomography (CT) is a vital diagnostic tool in clinical applications, widely used for disease screening and diagnosis. However, CT scans usually involve x-rays, which expose patients to radiation and potential health risks. To reduce these risks, several strategies have been developed to achieve low-dose CT imaging. These strategies include reducing the number of incident photons (low-milliampere-second), decreasing the sampling views (sparse-view), and limiting the sampling angles (limited-angle) during CT examinations. As a result, low-dose CT imaging often leads to reduced image quality, characterized by a lower signal-to-noise ratio and more severe noise-induced artifacts. This degradation in image quality can impact diagnosis accuracy, potentially rendering insufficient treatment for patients and increased risks.

In recent years, deep learning (DL) methods have emerged as powerful tools to reconstruct low-dose CT images and to enhance image quality [1–13]. Most methods directly learn high-quality CT images from low-dose sinograms/images using well-trained networks trained on large amounts of centralized data. These methods can be defined as centralized DL-based methods, where the training datasets require centralized collection from different vendors/clients. While these centralized DL-based methods show the potential for promising reconstruction performance, successful training requires large and diverse training data from different imaging geometries and scanning protocols. In CT imaging, obtaining sufficient centralized training data is challenging due to high economic and labor costs, as well as data privacy concerns. Moreover, data heterogeneity exists among different medical clients, with variations in imaging equipment, scanning parameters, protocols, and anatomical sites. This further degrades the training efficiency and generalization ability of centralized CT reconstruction models.

To overcome these limitations, federated learning (FL) has emerged as a promising machine learning paradigm, enabling model training across different clients without sharing data. FL-based methods learn a global model by averaging model parameters trained on each client’s private data and then broadcasting the global model back to each client for collaborative training. This can protect data privacy and reduce the need for central data collection. However, due to heterogeneous data distributions across clients (e.g., different imaging geometries and scanning protocols), sharing a global model can lead to substantial performance degradation and reduced training efficiency. Building on traditional FL-based frameworks [14–20], researchers have been developing FL-based CT reconstruction methods with special consideration of data heterogeneity across clients [21,22]. These methods aggregate parameters from local clients on a central server and mitigate the effect of data heterogeneity to some extent by aligning latent feature distribution across clients.

**Fig. 1.**
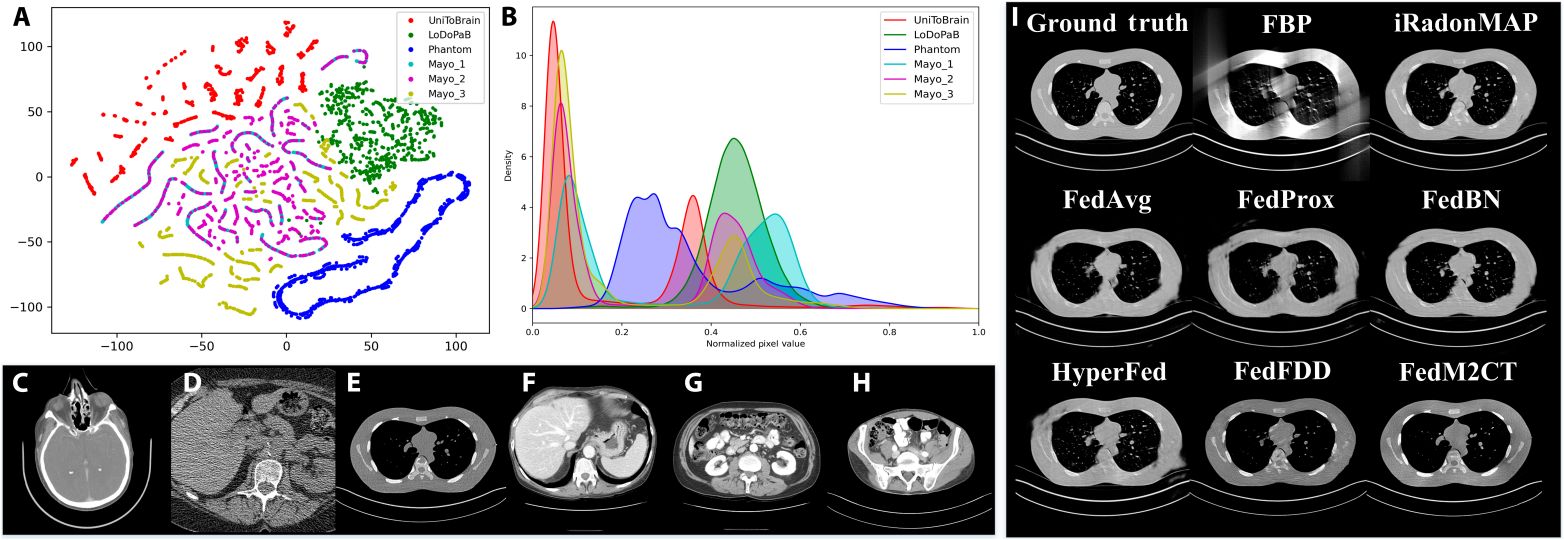
(A) The t-distributed stochastic neighbor embedding (t-SNE) visualization from 6 selective datasets. (B) The histogram dissimilarity. Example images of (C) the UniToBrain dataset, (D) the LoDoPaB dataset, (E) a physical phantom dataset, and (F to H) the Mayo clinic dataset. These datasets were obtained using different scanners and protocols. (I) In the case of severe data heterogeneity (i.e., both low-milliampere-second cases and limited-angle cases in the all-in-one reconstruction task), the limited-angle reconstruction performance of different competing methods. It can be seen that the traditional federated learning (FL) frameworks obtain degraded performance due to the severe data heterogeneity, while the proposed FedM2CT can produce promising results. FBP, filtered back projection; FedAvg, federated averaging; FedProx, federated learning with proximal terms; FedBN, federated learning with localized bulk normalization; HyperFed, personalized federated learning with hypernetworks; FedFDD, federated learning with frequency-domain decomposition.

However, the severe data heterogeneity can still seriously degrade reconstruction performance [23,24]. These methods can enhance image quality for various low-milliampere-second cases within a unified framework, but they fail to simultaneously support sparse-view samples and limited-angle samples. For example, Fig. 1 presents the t-distributed stochastic neighbor embedding results, quantitative differences in histograms, and corresponding example CT images from 6 datasets [25]. The datasets are from different imaging geometries and sampling protocols. It can be seen that the CT image distributions from different datasets are nonindependent and identically distributed due to variations in imaging geometries, scanning protocols, and subject cohort. Moreover, features from the same dataset are clustered together, while features from different datasets are well separated. As a result, we can see 4 distinct clusters of latent features. To quantify the data heterogeneity, Table 1 presents a heterogeneity assessment of the 6 CT datasets using Kullback–Leibler divergence and gray-level co-occurrence matrix (GLCM) features. The *P* values calculated via the Wilcoxon rank-sum test indicate statistical significance for pairwise differences, where *P* < 0.001 denotes a significant difference. Table 1 shows marked heterogeneity (all *P* < 0.001) among non-Mayo datasets, while Mayo data exhibit weaker divergence (*P* < 0.004), consistent with their clustered feature distribution in Fig. 1A. Due to severe data heterogeneity, simple averaging-based aggregation can lead to substantial performance degradation in the traditional FL frameworks, as shown in Fig. 1I [18].

**Table 1.** Quantitative heterogeneity assessment of 6 CT datasets using KL divergence and GLCM features. The *P* values (calculated via the Wilcoxon rank-sum test) indicate statistical significance for pairwise differences, and *P* < 0.001 denotes significant difference.

Dataset	UniToBrain	LoDoPaB	Phantom	Mayo_1	Mayo_2	Mayo_3
(a) Comparison with KL divergence across 6 CT datasets						
UniToBrain	ND	1.156	0.847	0.807	0.710	1.006
		*P* < 0.001	*P* < 0.001	*P* < 0.001	*P* < 0.001	*P* < 0.001
LoDoPaB	1.357	ND	0.497	0.266	0.245	0.400
	*P* < 0.001		*P* < 0.001	*P* < 0.001	*P* < 0.001	*P* < 0.001
Phantom	0.442	0.536	ND	0.267	0.264	0.363
	*P* < 0.001	*P* < 0.001		*P* < 0.001	*P* < 0.001	*P* < 0.001
Mayo_1	0.899	0.301	0.316	ND	0.002	0.263
	*P* < 0.001	*P* < 0.001	*P* < 0.001		*P* = 0.581	*P* < 0.001
Mayo_2	0.906	0.279	0.310	0.002	ND	0.276
	*P* < 0.001	*P* < 0.001	*P* < 0.001	*P* = 0.004		*P* < 0.001
Mayo_3	1.040	0.557	0.376	0.259	0.278	ND
	*P* < 0.001	*P* < 0.001	*P* < 0.001	*P* < 0.001	*P* < 0.001	
(b) Comparison with GLCM features across 6 CT datasets						
UniToBrain	ND	8.990	96.000	13.740	13.446	16.444
		*P* < 0.001	*P* < 0.001	*P* < 0.001	*P* < 0.001	*P* < 0.001
LoDoPaB	8.990	ND	87.009	22.730	22.436	25.434
	*P* < 0.001		*P* < 0.001	*P* < 0.001	*P* < 0.001	*P* < 0.001
Phantom	96.000	87.009	ND	0.267	0.264	0.363
	*P* < 0.001	*P* < 0.001		*P* < 0.001	*P* < 0.001	*P* < 0.001
Mayo_1	13.740	22.730	109.739	ND	0.294	2.704
	*P* < 0.001	*P* < 0.001	*P* < 0.001		*P* = 0.004	*P* < 0.001
Mayo_2	13.446	22.436	109.445	0.294	ND	2.998
	*P* < 0.001	*P* < 0.001	*P* < 0.001	*P* = 0.004		*P* < 0.001
Mayo_3	16.444	25.434	112.443	2.704	2.998	ND
	*P* < 0.001	*P* < 0.001	*P* < 0.001	*P* < 0.001	*P* < 0.001	

CT, computed tomography; KL, Kullback–Leibler; GLCM, gray-level co-occurrence matrix

Based on the observations, we propose a novel federated metadata-constrained method with mutual learning for all-in-one CT reconstruction, termed FedM2CT. The proposed FedM2CT consists of 3 modules: task-specific iRadonMAP (TS-iRadonMAP), condition-prompted mutual learning (CPML), and federated metadata learning (FMDL). For each client, TS-iRadonMAP is effectively trained on the local private dataset. Each client maintains a private model with customizable architectures, enabling information exchange with the server. During the local parameter-sharing phase, CPML facilitates efficient cross-client–server communication by integrating mutual learning between TS-iRadonMAP (local clients) and FMDL (server). This mechanism not only preserves client privacy but also enhances exchange efficiency. Similar to previous studies [24], the server stores high-quality metadata (e.g., including low-dose images and their corresponding normal-dose counterparts) from diverse imaging geometries and sampling protocols. FMDL subsequently trains a supervised metamodel using these metadata and then combines the metanetwork parameters with aggregated CPML parameters from all clients to alleviate data heterogeneity. Finally, FMDL broadcasts the unified parameters to all clients for the next round.

Furthermore, we integrate task-specific sampling conditions (e.g., imaging geometries and sampling protocols) into CPML and FMDL to dynamically modulate the reconstruction process. Consequently, FedM2CT employs CPML to ensure data privacy while enhancing training efficiency across heterogeneous datasets from multiple clients. We conducted comprehensive validation through extensive multivendor CT datasets to quantitatively and qualitatively evaluate FedM2CT’s reconstruction performance. Experimental results demonstrate that FedM2CT achieves superior performance across diverse CT reconstruction tasks, both qualitatively and quantitatively. FedM2CT holds the potential to achieve all-in-one CT reconstruction within the FL framework, aiming to substantially enhance image quality across diverse clinical situations.

Our contributions are 4-fold: (a) We design a practical FL situation with metadata on the server for low-dose CT imaging tasks. (b) We propose TS-iRadonMAP for client-side task-specific CT reconstruction and develop CPML to enable cross-client–server information exchange. (c) We encode task-specific sampling conditions into conditional parameter vectors via prompt-based adaptation, integrating these vectors into CPML and FMDL to guide adaptive reconstruction. (d) We conduct extensive experiments on 6 publicly available CT datasets and a private clinical dataset across 3 reconstruction tasks, demonstrating the superiority of FedM2CT in all-in-one reconstruction.

### Related work

#### Centralized DL-based CT reconstruction

In recent years, numerous centralized DL-based CT reconstruction methods have emerged. These methods collect diverse CT datasets acquired through different imaging geometries and sampling protocols for enhanced network training. Liang et al. [8] proposed an edge-enhancement-based densely connected convolutional neural network to reduce noise-induced artifacts in low-dose CT imaging. Marcos et al. [9] developed a deep neural network incorporating dilated convolution, batch normalization, rectified linear unit layers, and fused spatial-channel attention modules to enhance image quality. Subsequently, Gholizadeh-Ansari et al. [10] proposed a deep neural network employing dilated convolution with variable dilation rates instead of standard convolution to capture more contextual information in low-dose CT images.

Several studies have focused on cross-domain DL-based methods for low-dose CT reconstruction. Zhu et al. [11] proposed AUTOMAP, which uses multiple fully connected layers to achieve end-to-end image reconstruction. Kang et al. [12] introduced a wavelet-transform-based CT reconstruction algorithm that effectively reduces noise in low-dose CT images. Wang et al. [13] developed a learned-regularization-based CT reconstruction method to improve CT image quality. He et al. [2] proposed a DL-based CT reconstruction algorithm within the alternating direction method of multipliers (ADMM) framework, which adaptively learns model parameters to reconstruct high-quality images. Huang et al. [3] presented a cycle-consistent generative adversarial network to suppress noise-induced artifacts. Feng et al. [4] proposed a dual-residual convolutional neural network integrating sinogram-domain and image-domain feature extraction for low-dose CT image reconstruction. Ding et al. [5] introduced a method that adapts hyperparameters and employs a learnable image prior based on a framelet filter bank. Tao et al. [6] presented the view-by-view back-projection tensor (VVBP-Tensor) domain learning framework to reconstruct CT images at multiple dose levels. He et al. [1] also proposed the iRadonMAP algorithm to characterize the Radon inversion process via DL for high-quality CT reconstruction. However, all of these centralized DL-based methods rely on large amounts of diverse paired data, which are difficult to collect due to high costs and patient privacy concerns.

#### FL-based CT reconstruction

FL is a distributed collaborative framework designed for training models on decentralized data [19,26,27]. When interclient distribution characteristics exhibit marked heterogeneity, traditional FL-based methods (e.g., federated averaging [FedAvg] [19]) fail to achieve all-in-one reconstruction task by simplistic server-side averaging aggregation. Then, current FL-based CT reconstruction methods consider the heterogeneity of CT data across clients. For example, Yang et al. [21] proposed personalized federated learning with hypernetworks (HyperFed), a hypernetwork-based FL method for personalized CT imaging. HyperFed integrates a globally shared imaging network with institution-specific hypernetworks to address both local data adaptation and the global reconstruction task. Chen et al. [22] developed a personalized FL (federated learning with frequency-domain decomposition [FedFDD]) to enhance model generalization across clients by decomposing images into frequency components. FedFDD updates high-frequency signals in the FL setting while preserving local low-frequency characteristics. Wang et al. [28] introduced a peer-to-peer federated continual learning strategy to improve low-dose CT imaging performance across multiple institutions. Notably, traditional FL-based CT reconstruction frameworks typically lack server-side metadata integration, a major limitation for performance enhancement. To address this, Li et al. [23] constructed a standardized dataset of high-quality paired data on the server, which was utilized to further train the global model, stabilizing and standardizing feature learning. Chen et al. [24] developed the FedCG framework, which leverages server-side metadata to perform multiple reconstruction tasks and improve CT reconstruction performance. Although these methods have shown the potential to improve image quality across different imaging geometries and sampling protocols, there is still room for further improvement. First, all client models in the existing framework share the same architecture, lacking flexibility for different acquisition settings. Second, the direct exchange of model updates between clients and servers still poses privacy risks. Third, these methods have not yet comprehensively explored a wider range of imaging task situations. Unlike these methods, FedM2CT utilizes TS-iRadonMAP at clients and integrates server-side metadata-guided parameter adaptation and prompt-based mutual learning into the framework, mitigating the effect of data heterogeneity.

## Methods

### Overview of FedM2CT

**Fig. 2.**
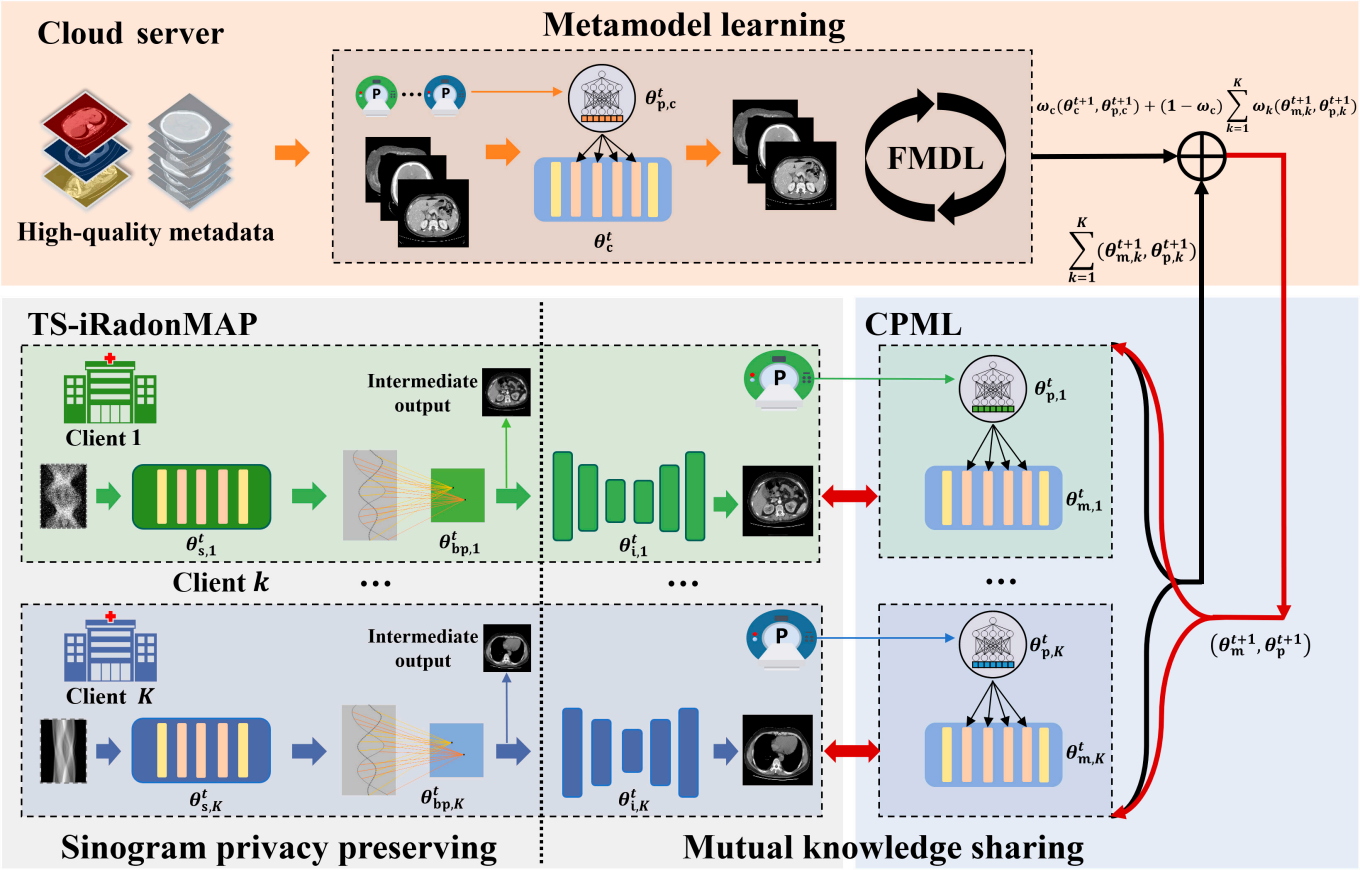
Overall architecture of FedM2CT for all-in-one CT reconstruction. FedM2CT consists of 3 modules, i.e., task-specific iRadonMAP (TS-iRadonMAP), condition-prompted mutual learning (CPML), and federated metadata learning (FMDL). TS-iRadonMAP performs the local CT image reconstruction task using a private model with any architecture, facilitating information exchange with the server. CPML is designed to share the knowledge in the local parameter-sharing submodule in TS-iRadonMAP via mutual learning. High-quality metadata are collected on the server to train the metamodel. FMDL adaptively combines the parameters of the metamodel on the server with the parameters of CPML to mitigate the effect of data heterogeneity. In particular, the imaging geometry and scanning protocol parameters of each client are employed to modulate the client-specific reconstruction task.

All-in-one CT reconstruction from multiple imaging geometries and sampling protocols within one model is a challenging task for the existing centralized DL-based CT reconstruction methods. In this work, we develop a federated metadata-constrained iRadonMAP framework with mutual learning (FedM2CT). Fig. 2 shows the overall architecture of FedM2CT for all-in-one CT reconstruction. FedM2CT consists of 3 modules, i.e., TS-iRadonMAP, CPML, and FMDL. TS-iRadonMAP performs the local CT image reconstruction task using a private model with any architecture, facilitating information exchange with the server. CPML is designed to share the knowledge in the local parameter-sharing submodule in TS-iRadonMAP via mutual learning. High-quality metadata are collected on the server to train the metamodel. FMDL adaptively combines the parameters of the metamodel on the server with the parameters of CPML to mitigate the effect of data heterogeneity. The following part of this section details the design of each module.

### Task-specific iRadonMAP

In the proposed FedM2CT, various CT reconstruction models can be selected as the backbone network. The iRadonMAP operates as a dual-domain DL framework for CT reconstruction by synergistically processing projection-domain sinograms and image-domain data through 3 interconnected modules: (a) a fully connected filtering module that extracts projection-domain features via parallel convolutional layers, (b) a sinogram back-projection module that reconstructs initial images using inverse Radon transform principles, and (c) a universal network module that refines residual features across domains [1]. Then, iRadonMAP is chosen as the backbone network $\Phi_{\text{dual}}$ with parameters θ, which performs the mapping of sinogram x to image $y^{*}$ via the dual-domain network as follows:

$$y^{*}=\Phi_{\text{dual}}\left(x;\;\theta \right)=\Phi_{i}\left\{\Phi_{\mathrm{bp}}\left[\Phi_{s}\left(x;\;\theta_{s}\right);\;\theta_{\mathrm{bp}}\right];\;\theta_{i}\right\}$$ (1)

where $\Phi_{\text{dual}}$ consists of 3 main components: the sinogram-domain sub-network $\Phi_{s}$ with parameters $\theta_{s}$, the back-projection learnable module $\Phi_{\mathrm{bp}}$ with parameters $\theta_{\mathrm{bp}}$, and the image-domain sub-network $\Phi_{i}$ with parameters $\theta_{i}$. Specifically, $\Phi_{\mathrm{bp}}$ is determined by the CT imaging geometry, scanning protocols, and other factors, making iRadonMAP sensitive to these variations. Therefore, this sensitivity should be addressed through TS-iRadonMAP. In this context, TS-iRadonMAP performs the personalized dual-domain CT reconstruction task (i.e., low-milliampere-second, sparse-view, or limited-angle) for each client, employing the architecture of $\Phi_{s}$ and $\Phi_{i}$ based on the characteristics of the client-specific CT data. To address the privacy preservation concerns and heterogeneous effects in the CT imaging field, TS-iRadonMAP introduces a sinogram privacy-preserving strategy, which allows for processing task-specific sinograms without sharing $\theta_{s}$ and $\theta_{\mathrm{bp}}$. The final CT image is reconstructed by $\Phi_{i}$, with its parameters $\theta_{i}$ dynamically updated via CPML to optimize reconstruction quality.

### Condition-prompted mutual learning

**Fig. 3.**
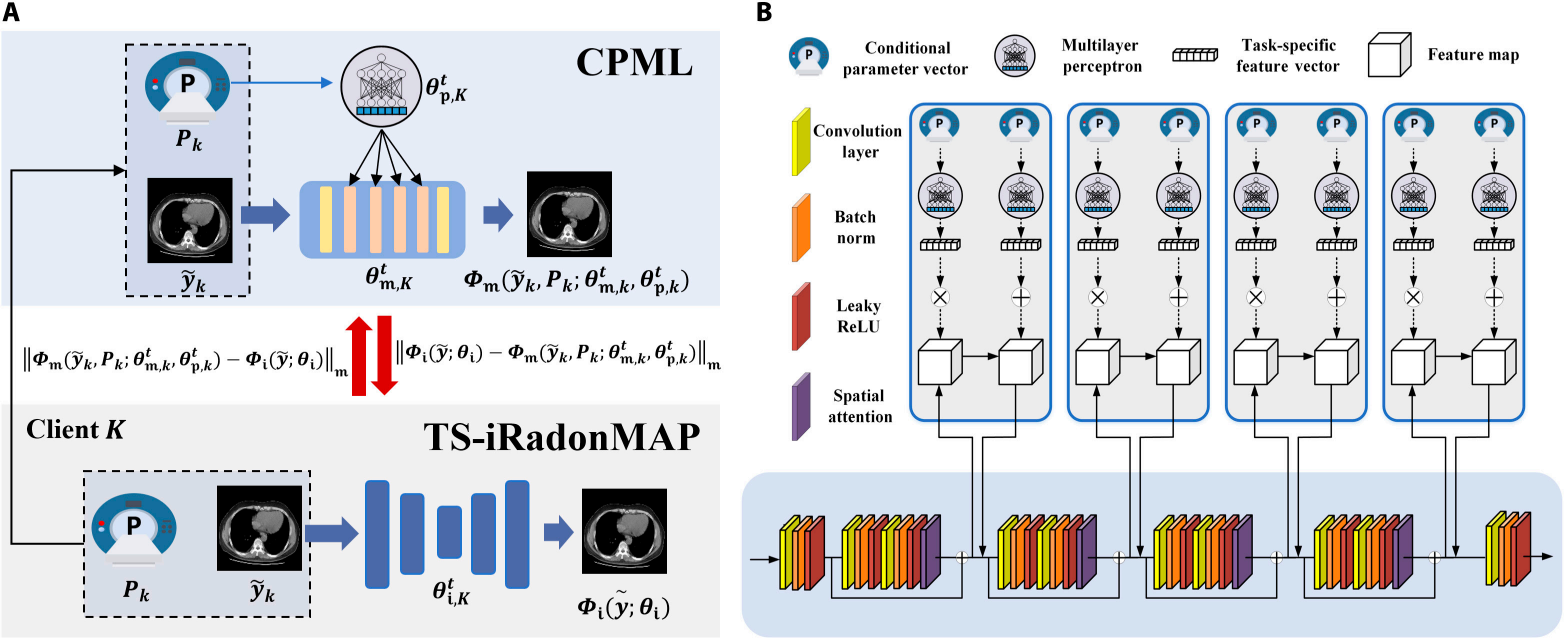
Overall architecture of CPML, including (A) mutual knowledge sharing and (B) conditional prompt. CPML allows indirectly exchanging latent critical information between local clients and the server, which helps improve exchange efficiency via mutual learning and protect client privacy without directly transmitting the local knowledge to the server. The imaging geometry and scanning protocol parameters of each client are employed to modulate the client-specific reconstruction task via conditional prompt.

As shown in Fig. 3, to exchange the information across the TS-iRadonMAP of each client, CPML deploys a shared model $\Phi_{m}$ at each client during the local parameter-sharing stage. In this work, we introduce a deep mutual learning knowledge distillation technique [29] into the CT reconstruction, enabling CPML to exchange knowledge of local data. $\Phi_{m}$ asynchronously receives intermediate outputs $\tilde{y}$ from TS-iRadonMAP (i.e., image outputs from $\Phi_{\mathrm{bp}}$) and learns local data distribution mutually with the $\Phi_{i}$ at each client. In each round of mutual training updates, we adopt the Huber distance as the mutual learning loss to dynamically constrain the output consistency of the 2 models, which can be defined as follows:

$$\begin{array}{c} {\left\|\Phi_{i}\left(\tilde{y};\;\theta_{i}\right)-\Phi_{m}\left(\tilde{y};\;\theta_{m}\right)\right\|}_{m}=\left\{\begin{array}{cc} 0.5\cdot a^{2}, & a\leq \delta \\ \delta \cdot \left|a\right|-0.5\cdot \delta^{2}, & a>\delta \end{array}\right.\\ \text{where}\;a=\Phi_{i}\left(\tilde{y};\;\theta_{i}\right)-\Phi_{m}\left(\tilde{y};\;\theta_{m}\right) \end{array}$$ (2)

where the control hyperparameter δ is experimentally set to 0.1. For mutual knowledge sharing, $\Phi_{i}$ and $\Phi_{m}$ are synchronized to update parameters with reconstruction loss and mutual learning loss. Then, CPML transmits the shared parameters $\theta_{m}$ of each client to the server, acting as a proxy of TS-iRadonMAP. The network architecture of $\Phi_{\text{m}}$ can employ lightweight optimization for communication efficiency while preserving architectural consistency with the metamodel $\Phi_{c}$. Therefore, this can mitigate aggregation-induced destructive impacts during parameter synchronization while protecting data privacy, by avoiding the direct sharing of the local parameters from TS-iRadonMAP.

In addition, based on the conditional prompt method, task-specific imaging geometries and sampling protocols are introduced to modulate $\Phi_{m}$ and $\Phi_{c}$. This process guides the network to learn task-specific features at each client, as shown in Fig. 3B. Inspired by previous studies [13,30], various scanning parameters are first concatenated to obtain an initial vector. Then, for parameters with large magnitudes, they are logarithmized to reduce their scale. Finally, normalization is performed to ensure that the parameters are scaled to a consistent range. Consequently, both the imaging geometry and sampling protocol parameters are characterized by a conditional parameter vector P as follows:

$$P={\left[g_{1},\cdots ,\;g_{n},\;G,\;R\right]}^{T}$$ (3)

where g denotes CT imaging geometry parameters, such as source-to-detector distance, source-to-center distance, projection views, number of detector bins, and detector bin size. G is a scalar parameter defining the sampling condition, i.e., x-ray intensities and sampling views. R is a customizable scalar that indicates the anatomical region, such as the brain, chest, and abdomen. Then, P is encoded into a high-dimensional vector by a shallow conditional prompted network $\Phi_{p}$ (i.e., multilayer perceptron), which is implemented as

$$V_{1},V_{2}=\Phi_{p}\left(P;\;\theta_{p}\right)$$ (4)

where $\theta_{p}$ denotes the $\Phi_{p}$ parameters. $V_{1}$ and $V_{2}$ are the outputs of the shallow network, which denote the learned posterior information under task-specific imaging. Then, $V_{1}$ and $V_{2}$ prompt the feature map of specific networks layer as follows:

$$\tilde{f}=V_{1}\times f+V_{2}$$ (5)

where f is the feature map of network layer and $\tilde{f}$ is the prompted feature map. CPML make $\Phi_{m}$ and $\Phi_{c}$ adaptive to the data distribution. Therefore, FedM2CT enables personalized reconstruction for datasets with different sampling conditions across multiple vendors and clients.

### Federated metadata learning

Unlike the traditional FL framework that employs average aggregation schemes, FedM2CT contains a large amount of high-quality metadata $\left(\mu ,\;\mu^{*}\right)$ on the server. This strategy aligns closely with real-world application situations and has been used to investigate data heterogeneity and generalization ability [23,24]. The local data at each client come from a single source, which is obtained from one specific scanner or one hospital. In contrast, $\left(\mu ,\;\mu^{*}\right)$ is highly diverse, which is derived from publicly available datasets. It contains paired low-dose images and normal-dose images generated under a diverse range of random scan protocols, some of which match those used by the clients themselves. Then, metadata are used to train metamodel $\Phi_{c}\left(\theta_{c},\;\theta_{p,c}\right)$ with one extra training round. Similar to CPML, both the imaging geometry and sampling protocol parameters are prompted by a conditional parameter vector P. After the metamodel training on the server, $\left(\theta_{c},\;\theta_{p,c}\right)$ are aggregated with $\theta_{m}$ uploaded by each client in a weighted manner.

### Training process

Specifically, the training process of FedM2CT, as summarized in Algorithm 1 and Fig. 2, can be outlined in the following steps:

1. Local TS-iRadonMAP training: At each training round t∈T, TS-iRadonMAP at the kth client is trained using the local training dataset, which includes a low-dose sinogram $x_{k}$ and normal-dose images $y_{k}^{*}$:

$$\begin{array}{c} \begin{array}{c} \theta_{k}^{t+1}=\underset{\theta_{k}^{t}}{\text{arg min}}{\left\|y_{k}^{*}-\Phi_{\text{dual}}\left(x_{k};\;\theta_{k}^{t}\right)\right\|}_{2}+\varepsilon_{k}{\left\|\Phi_{\text{dual}}\left(x_{k};\;\theta_{k}^{t}\right)\right\|}_{\mathrm{TV}}\\ \;+\alpha_{k}{\left\|\Phi_{i}\left({\tilde{y}}_{k};\;\theta_{i,k}^{t}\right)-\Phi_{m}\left({\tilde{y}}_{k},\;P_{k};\;\theta_{m,k}^{t},\;\theta_{p,k}^{t}\right)\right\|}_{m} \end{array} \end{array}$$ (6)

where $y_{k}^{*}$ represents normal-dose CT images as labels; $\theta_{k}^{t}=\left(\theta_{s,k}^{t},\;\theta_{bp,k}^{t},\;\theta_{i,k}^{t}\right)$ are all network parameters; ${\left\|\cdot \right\|}_{2}$ denotes the mean squared error loss function, which serves as the data fidelity term; and ${\left\|\cdot \right\|}_{TV}$ is the total variation constraint (TV). The parameter $\varepsilon_{k}$, a control hyperparameter for unpaired data, is set to zero for paired data, and $\alpha_{k}$ balances the mutual learning loss.

2. Mutual knowledge sharing: Meanwhile, in CPML, the $\Phi_{m}$ at each client receive $\tilde{y}$ from $\Phi_{bp}$ for model updating to share knowledge mutually with the $\Phi_{i}$ of TS-iRadonMAP, which can be defined as follows:

$$\begin{array}{c} \begin{array}{c} \begin{array}{c} \begin{array}{c} \left(\theta_{m,k}^{t+1},\theta_{p,k}^{t+1}\right)=\underset{\left(\theta_{m,k}^{t},\theta_{p,k}^{t}\right)}{\text{arg}\;\text{min}}{\left\|y_{k}^{*}-\Phi_{m}\left({\tilde{y}}_{k},\;P_{k};\;\theta_{m,k}^{t},\;\theta_{p,k}^{t}\right)\right\|}_{2}+\varepsilon_{k}{\left\|\Phi_{m}\left({\tilde{y}}_{k},\;P_{k};\;\theta_{m,k}^{t},\;\theta_{p,k}^{t}\right)\right\|}_{TV}\\ \;+\left(1-\alpha_{k}\right){\left\|\Phi_{m}\left({\tilde{y}}_{k},\;P_{k};\;\theta_{m,k}^{t},\;\theta_{p,k}^{t}\right)-\Phi_{i}\left({\tilde{y}}_{k};\;\theta_{i,k}^{t}\right)\right\|}_{m}. \end{array} \end{array} \end{array} \end{array}$$ (7)

3. Federated metadata training: Then, $\theta_{c}$ is updated by low-dose images μ and the corresponding normal-dose images $\mu^{*}$:

$$\left(\theta_{c}^{t+1},\theta_{p,c}^{t+1}\right)=\underset{\left(\theta_{c}^{t},\theta_{p,c}^{t}\right)}{\text{arg}\;\text{min}}{\left\|\mu^{*}-\Phi_{c}\left(\mu ,\;P;\;\theta_{c}^{t},\;\theta_{p,c}^{t}\right)\right\|}_{2}$$ (8)

4. Uploading, aggregating, and broadcasting: Once the metamodel and TS-iRadonMAP are well trained, $\left(\theta_{m,k}^{t+1},\;\theta_{p,k}^{t+1}\right)$ are uploaded to the server. Then, the server aggregates them with $\left(\theta_{c}^{t+1},\;\theta_{p,c}^{t+1}\right)$ for global model updating as follows:

$$\left(\theta_{c}^{t+1},\theta_{p,c}^{t+1}\right)=\omega_{c}\left(\theta_{c}^{t+1},\theta_{p,c}^{t+1}\right)+\left(1-\omega_{c}\right)\sum_{k=1}^{K}\omega_{k}\left(\theta_{m,k}^{t+1},\theta_{p,k}^{t+1}\right),$$ (9)

where $\omega_{c}$ is the aggregated weight of $\Phi_{c}$, $\omega_{k}$ is the aggregated weight of $\Phi_{m}$ for each client, and K is the total number of clients. Finally, the cloud server broadcasts $\left(\theta_{c}^{t+1},\theta_{p,c}^{t+1}\right)$ back to CPML to efficiently exchange information with $\Phi_{i,k}$.


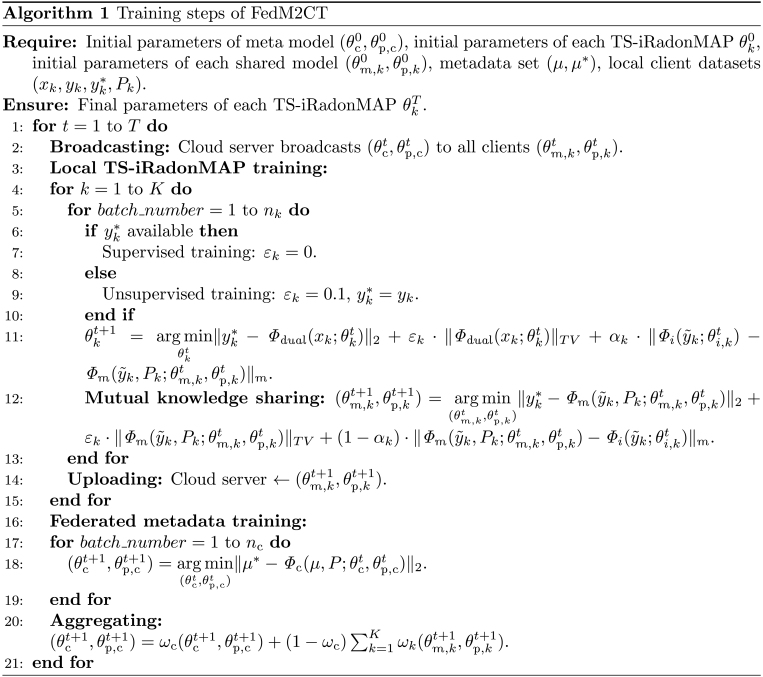


### Network architecture

TS-iRadonMAP consists of a sinogram-domain sub-network, a back-projection learnable module, and an image-domain sub-network. Specifically, the sinogram-domain sub-network remains retained locally without participating in federated parameter sharing. Empowered by CPML, the image-domain sub-network achieves architecture-agnostic knowledge exchange with the shared model. The client-specific architectures are required only to maintain identical input and output dimensions. This design enables our method to flexibly customize both sub-networks as either different architectures or depth variants of the same architecture, depending on client-specific CT data characteristics (e.g., noise profiles and anatomical regions) and reconstruction requirements (e.g., resolution-demanding, computation-constrained situations).

The sinogram-domain sub-network utilizes a ResNet architecture, where the first 2 layers are 2-dimensional convolutional layers with kernel sizes of 1 × 3 that map the sinogram data into a multichannel feature map. Each convolution is followed by group normalization and leaky ReLU activation. Subsequently, there are 4 sinogram filter units constructed from 3 residual convolution blocks with channel attention layers. The 2 convolutional output layers of the sinogram-domain sub-network merge the high-dimensional feature maps back into the sinogram-domain data. The convolution kernel size is set to 1 × 3 and filled with zeros to approximate the filtering process of the traditional filtered back-projection (FBP) method. The back-projection layer implements a differentiable operator based on the virtual CT system’s geometry, utilizing the ASTRA toolbox [31] for FBP computation. Though parameter-free, its gradient is computed through virtual forward projection during backpropagation. The backward pass employs ASTRA’s line integral projection to approximate the Jacobian of the back-projection operation. This design ensures end-to-end trainability while preserving physical consistency with CT reconstruction principles. The image-domain sub-network utilizes a U-Net architecture with a 4-layer depth for both downsampling and upsampling paths. Each downsampling path progressively reduces the spatial resolution and extracts features through multiple convolutional blocks. Each convolutional block contains two 3 × 3 convolutional layers, a leaky ReLU activation, and 2 × 2 strided convolutions. The upsampling path incrementally restores the spatial resolution through multiple transposed convolutional layers and convolutional blocks, connecting to the feature map of the corresponding layer of the downsampling path. Finally, the feature maps are converted to output maps by a 3 × 3 convolutional layer.

In CPML, the network architecture of the shared model is intentionally designed to be relatively lightweight and it consists of 2 modules: ResNet and the conditional prompt network. Similar to the sinogram-domain sub-network, the ResNet module has 4 image enhancement units in the middle of the convolutional layer. In addition, these image enhancement units are constructed from residual convolutional blocks with spatial attention, and the size of the convolutional kernel is set to 7 × 7. The conditional prompt network is composed of conditional mapping units wherein each unit has 3 fully connected layers encoding the conditional parameter vectors to guide the output of each layer of ResNet.

## Results

### Datasets

To validate and evaluate the reconstruction performance of FedM2CT, we collected 6 publicly available datasets and 1 private clinical dataset. A total of 110,000 CT images were collected as the metadata for the cloud server from 6 publicly available datasets, namely, (a) 2016 NIH-AAPM-Mayo Clinic Low Dose CT Grand Challenge Dataset [32], which provides 12,886 CT images from 10 patients, acquired at 80 to 120 kVp and 200 effective mAs (Siemens SOMATOM Definition Flash/AS+); (b) Low-Dose CT Image and Projection Dataset [33], which includes 19,916 CT images from patient exams acquired at 120 kVp and 250/300 effective mAs (Siemens SOMATOM Definition Flash/AS+, GE Discovery CT 750 HD); (c) UniToBrain Dataset [34], comprising 19,694 brain CT perfusion images from 100 patients acquired at 80 kVp and 200 effective mAs (GE LightSpeed VCT); (d) AbdomenCT-1K Dataset [35], offering 25,946 CT scans acquired at multiple levels of kilovolt peaks and effective milliampere-seconds using scanners from GE, Philips, and Siemens; (e) CTSpine1K Dataset [36], which consists of 21,558 spinal CT images acquired at multiple levels of kilovolt peaks and effective milliampere-seconds using scanners from GE, Philips, Siemens, and Toshiba; and (f) LoDoPaB-CT Dataset [37], which contains 10,000 CT images of size 362 × 362 from 800 patients selected from the Lung Image Database Consortium (LIDC) and Image Database Resource Initiative (IDRI) database, acquired by different vendors at tube currents ranging from 40 to 627 mA.

With the approval of the Medical Ethics Committee of the local institute, we collected CT images from 6 different scanners from one local hospital. Details of the dataset are as follows: Data 1 contains 2,102 brain perfusion CT scans of 30 patients at 80 kVp and 100 validated mAs (GE Discovery CT 750 HD). Data 2 contains 1,954 CT images from 616 chest scans acquired at 120 kVp and 220 effective mAs (Philips MX 16-slice Brilliance CT). Data 3 contains 1,187 CT images of the chest and abdomen from 25 patients, acquired at 140 kVp and 280 effective mAs (GE LightSpeed16). Data 4 contains 1,598 chest CT images of 29 patients acquired at 110 kVp and 240 effective mAs (Siemens Emotion 16). Data 5 contains 1,603 physical phantom CT images acquired at 120 kVp and 400 effective mAs (Siemens SOMATOM Definition AS+). Data 6 contains 1,567 chest and abdominal CT images of 14 patients acquired at 140 kVp and 280 active mAs (GE LightSpeed16). Data 7 was acquired with Data 4 with different scan parameters settings. Data 8 was acquired with the LoDoPaB-CT subdataset. Data 9 was acquired with data 3 by setting different scan parameters.

All of the CT images are treated as normal-dose images and can be used to simulate low-dose CT sinograms with different imaging geometries and sampling protocols [38]. Table 2 shows details of the imaging geometry and scanning protocol parameters for all client datasets. The metadata are used to generate low-dose metadata for 3 different situations: the low-milliampere-second-only situation (metadataset 1); the low-milliampere-second, sparse-view, and limited-angle situation with paired data (metadataset 2); and the low-milliampere-second situation without paired data (metadataset 3).

**Table 2.** Imaging geometry and scan protocol parameters for the different datasets

Parameter	Situation 1			Situation 2			Situation 3		
	Data 1	Data 2	Data 3	Data 4	Data 5	Data 6	Data 7	Data 8	Data 9
Data type	Paired	Paired	Paired	Paired	Paired	Paired	Paired	Unpaired	Unpaired
Number of voxels	512 × 512	512 × 512	512 × 512	512 × 512	512 × 512	512 × 512	512 × 512	362 ∗ 362	512 ∗ 512
Number of projection views	896	512	768	896	512	768	896	512	512
Number of detector bins	1,008	1,024	904	1,008	1,024	904	1,008	1,024	904
Length of a detector bin/mm	0.5480	0.6500	0.6000	0.5480	0.6500	0.6000	0.6500	0.6500	0.5480
Length of a voxel/mm	0.7421	0.7500	0.7000	0.7421	0.7500	0.7000	0.7500	0.7500	0.7421
DSD/mm	800.0	946.7	750.2	800.0	946.7	750.2	750.2	750.2	865.2
DSO/mm	550.0	476.8	538.5	550.0	476.8	538.5	476.8	476.8	613.0
Anatomical region (*A*)	$A_{\text{brain}}=1$	$A_{\text{chest}}=3$	$A_{\text{abdomen}}=2$	$A_{\text{chest}}=3$	$A_{\text{phantom}}=4$	$A_{\text{abdomen}}=2$	$A_{\text{abdomen}}=2$	$A_{\text{chest}}=3$	$A_{\text{abdomen}}=2$
Low-dose protocol (*G*)	X-ray intensity: 2 × 10^5^	X-ray intensity: 3 × 10^5^	X-ray intensity: 1 × 10^5^	X-ray intensity: 5 × 10^4^	Sampling angle: 2/3*π*	Sampling views: 96	X-ray intensity: 2 × 10^5^	X-ray intensity: 3 × 10^5^	X-ray intensity: 3 × 10^5^
Reconstruction filter	Ram-Lak	Ram-Lak	Ram-Lak	Shepp–Logan	Ram-Lak	Hamming	Ram-Lak	Ram-Lak	Ram-Lak

DSD, distance between the source and the detector; DSO, distance between the source and the rotation center

### Comparison methods

To evaluate the reconstruction performance of FedM2CT, we compare it with 6 different competing methods: filtered back-projection algorithm with a ramp filter kernel (FBP) [39], dual-domain reconstruction method (iRadonMAP) [1], FedAvg [19], federated learning with proximal terms (FedProx) [14], federated learning with localized bulk normalization (FedBN) [15], HyperFed [21], and FedFDD [22]. For a fair comparison, all methods employ the same reconstruction network, and each client in FedM2CT uses the same network architecture with the same number of layers. FedFDD methods are not originally designed for dual-domain reconstruction tasks and thus cannot be directly applied. To adapt it to our task, we introduce 2 image-domain sub-networks to separately process high- and low-frequency information. Moreover, peak signal-to-noise ratio (PSNR), structural similarity index measurement (SSIM), root-mean-square error (RMSE), learned perceptual image patch similarity (LPIPS), and Fréchet inception distance (FID) are used to assess the reconstruction accuracy.

### Implementation details

In this work, we conduct experiments for 3 different situations: Situation 1: low-milliampere-second CT reconstruction situation with different imaging geometries and scan protocols. To mitigate the effect of data heterogeneity with various CT imaging geometries and dose levels, we utilized data 1 to 3 for clients 1 to 3 and metadataset 1 in the experiments. Situation 2: low-dose CT reconstruction with different sampling conditions (i.e., low-milliampere-second, limited-angle, and sparse-view) and different imaging geometries. In the experiment, data 4 to 6 for clients 4 to 6 and metadataset 2 are utilized. Situation 3: low-milliampere-second CT reconstruction situation with unpaired and paired data. To tackle the issue of imaging task specificity, data 7 to 9 for clients 7 to 9 and metadataset 3 are utilized. For data 1 to 9, we randomly divide those datasets into 85% for training, 5% for validation, and 10% for testing.

During training, both the server and the local client perform a total of 200 rounds of aggregation, with 100 iterations per round for each TS-iRadonMAP and shared model and 200 iterations for the metamodel. Hyperparameters $\varepsilon_{k}$ and $\alpha_{k}$ were empirically set to 0.1 and 0.5, respectively, while $\omega_{c}$ was set to 0.55. The learning rate was set to 1 × 10^−4^ for the metamodel and 2 × 10^−5^ for the TS-iRadonMAP and shared models. All models were optimized using the Adam algorithm, with a momentum of 0.9 and a weight decay of 1 × 10^−4^. Forward and backward projections were performed using ASTRA toolbox v2.1.0 [31]. All DL models were implemented using PyTorch toolbox v1.8.0 with CUDA 11.1 [40] and trained on an NVIDIA RTX 4090 graphics processing unit with 24 GB of memory.

### CT reconstruction performance in situation 1

**Fig. 4.**
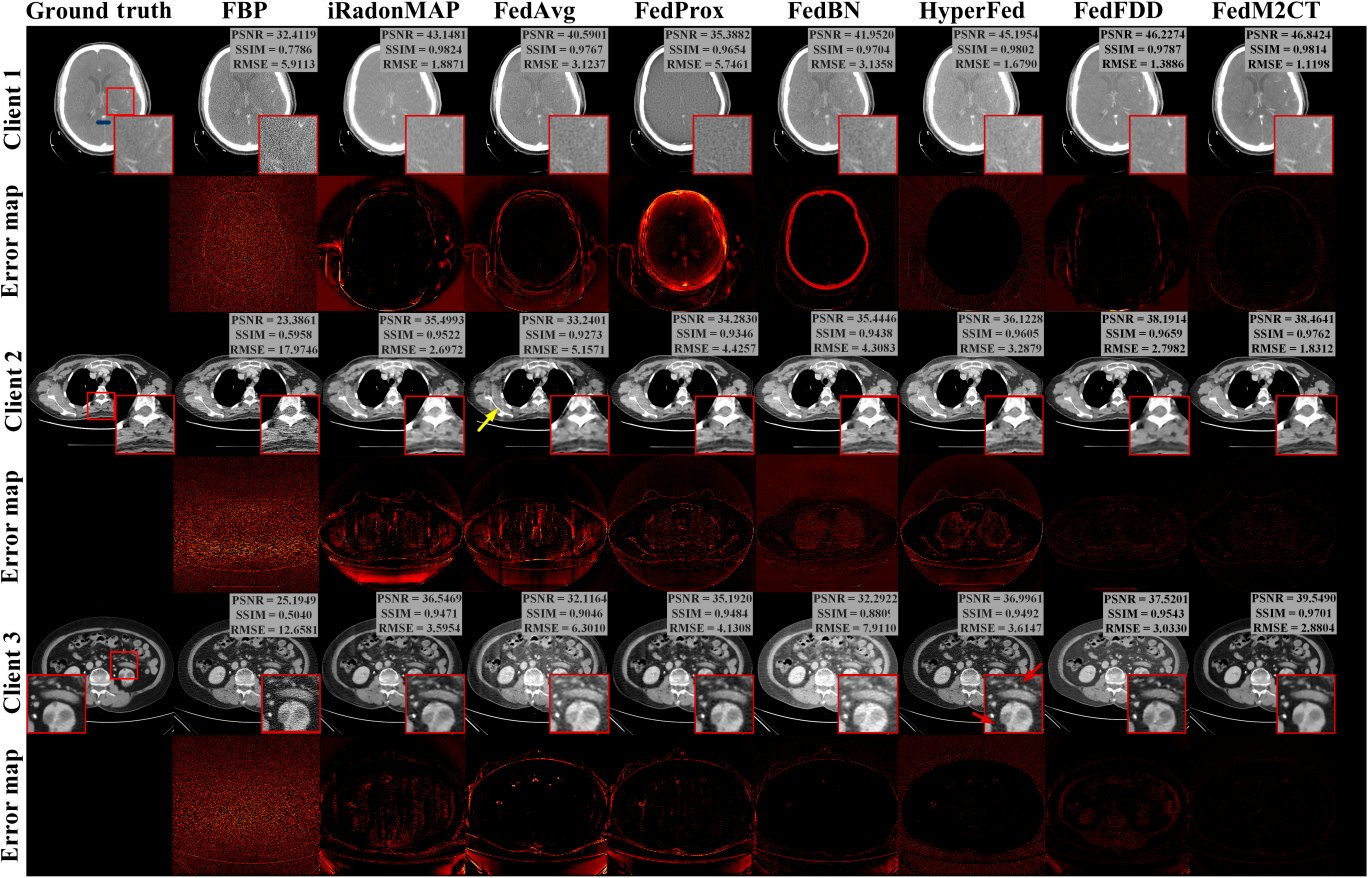
Visual comparisons of the representative images reconstructed by different methods from 3 clients in situation 1. Zoomed-in regions of interest (ROIs) highlighted by the red boxes are shown on the corresponding images. Quantitative measurements, including peak signal-to-noise ratio (PSNR), structural similarity index measurement (SSIM), and root-mean-square error (RMSE) values, are also listed. The display windows are [−160, 240] HU for all images and the zoomed-in ROIs.

Fig. 4 shows the comparison of reconstructed CT images using the proposed FedM2CT against other competing methods for situation 1. It is obvious that the images reconstructed by the traditional method FBP still suffer from severe noise-induced artifacts in all cases, while iRadonMAP trained with local datasets can produce CT images with reduced noise-induced artifacts at each client. The previously developed FL-based methods can suppress noise-induced artifacts to some extent with the help of FL. However, they still fail to accurately reconstruct finer structural details essential for high-quality CT imaging. For example, at client 1, FedProx produces underestimated values, especially in the zoomed-in region of interest (ROI) indicated by the red box. At client 2, there still exist undesired artifacts, which can be considered a false negative of defects indicated by the yellow arrows in the FedAvg-reconstructed images. At client 3, both FedBN and HyperFed distort the reconstructed images with undesired artifacts and value shift as shown in the zoomed-in ROIs. The failure of these existing FL-based methods in the all-in-one reconstruction task is due to the critical data heterogeneity and different noise distributions among all low-dose measurements, which impair the training efficiency of the traditional FL-based methods. Although FedFDD mitigates this issue through frequency-domain decomposition, its performance remains suboptimal under heterogeneous data conditions. While clients 1 and 2 produce accurate reconstructions, client 3’s output exhibits residual noise and insufficient structural details. Furthermore, FedM2CT enables superior reconstruction with lower errors, better noise-induced artifact suppression, and better structural detail recovery as compared to the other competing methods. This improvement is due to the metadata on the server and task-specific reconstruction, which allows FedM2CT to leverage critical latent features to enhance CT image quality at each client. Moreover, quantitative assessments (PSNR, RMSE, and SSIM values) demonstrate that FedM2CT consistently achieves superior performance compared to the competing methods across all clients, regardless of the evaluation metric used.

**Fig. 5.**
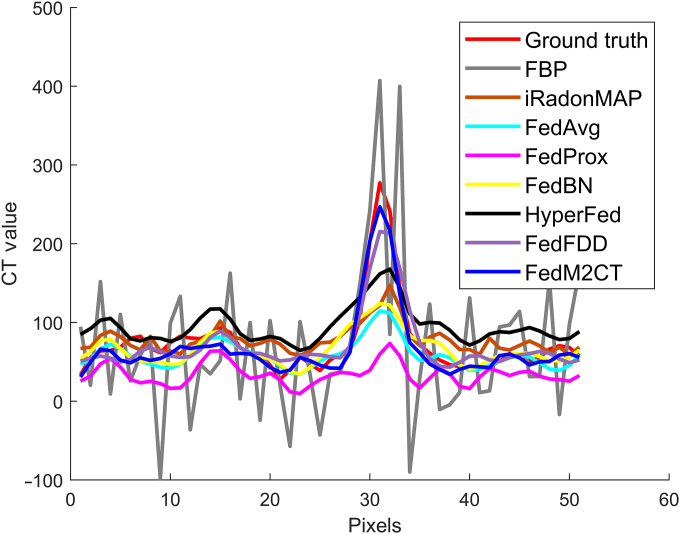
Profile comparisons among the different methods as indicated by the blue line at client 1 in Fig. 4.

Fig. 5 shows the profile comparison of the different methods, as indicated by the blue line in Fig. 4. The corresponding results show that the proposed FedM2CT provides more accurate reconstruction compared to the other competing methods. Table 3 summarizes the average PSNR, SSIM, and RMSE scores and standard deviations on the whole testing set of all of the methods from the 3 clients. The objective metrics in Table 3 indicate that FedM2CT produces the lowest reconstruction errors and the highest structural similarity to ground truth among all of the FL-based methods. Furthermore, *P* values and 95% confidence intervals (CIs) derived from the Wilcoxon signed-rank test are used to evaluate the statistical significance of each method’s improvement over FBP. The CIs confirm that for all metrics, FedM2CT’s differences relative to FBP are statistically significant, highlighting its superior performance. This quantitative result is consistent with the visual inspections mentioned above.

**Table 3.** Quantitative measures (mean ± standard deviation) of the reconstructions for different methods in situation 1 and situation 2, including *P* values and 95% confidence intervals.

Client	Method	PSNR		SSIM		RMSE	
		Situation 1	Situation 2	Situation 1	Situation 2	Situation 1	Situation 2
1st client	FBP	30.6918 ± 1.3231	25.8413 ± 1.4721	0.6832 ± 0.0822	0.6213 ± 0.1013	6.5883 ± 0.9982	12.0335 ± 1.1294
	iRadonMAP	45.5383 ± 2.0242	36.9607 ± 3.6187	0.9815 ± 0.0018	0.9179 ± 0.0034	1.4443 ± 0.4841	1.8584 ± 0.8805
		*P* < 0.001, [14.76, 15.67]	*P* < 0.001, [10.50, 11.87]	*P* < 0.001, [0.28, 0.31]	*P* < 0.001, [0.27, 0.32]	*P* < 0.001, [−5.51, −5.03]	*P* < 0.001, [−10.38, −9.94]
	FedAvg	40.8691 ± 5.3667	30.9336 ± 6.0061	0.9754 ± 0.0081	0.8776 ± 0.0123	3.6781 ± 1.3444	3.9645 ± 2.6386
		*P* < 0.001, [9.31, 11.60]	*P* < 0.001, [3.10, 5.56]	*P* < 0.001, [0.28, 0.31]	*P* < 0.001, [0.23, 0.27]	*P* < 0.001, [−3.05, −2.30]	*P* < 0.001, [−8.37, −7.26]
	FedProx	36.1468 ± 5.1978	33.7269 ± 5.3159	0.9609 ± 0.0192	0.8991 ± 0.0180	5.7851 ± 3.8707	3.9381 ± 3.8994
		*P* < 0.001, [4.76, 6.78]	*P* < 0.001, [7.04, 9.31]	*P* < 0.001, [0.26, 0.29]	*P* < 0.001, [0.25, 0.30]	*P* = 0.17, [−1.35, 0.38]	*P* < 0.001, [−8.66, −6.87]
	FedBN	42.2417 ± 5.1211	37.7382 ± 5.7886	0.9713 ± 0.0047	0.9148 ± 0.0142	3.6345 ± 2.1402	2.3722 ± 2.1852
		*P* < 0.001, [10.84, 13.05]	*P* < 0.001, [11.28, 13.31]	*P* < 0.001, [0.27, 0.30]	*P* < 0.001, [0.27, 0.31]	*P* < 0.001, [−2.87, −2.01]	*P* < 0.001, [−10.54, −9.66]
	HyperFed	45.5429 ± 3.0483	38.1944 ± 3.9665	0.9789 ± 0.0037	0.9317 ± 0.0223	1.7424 ± 0.7683	1.8202 ± 0.8651
		*P* < 0.001, [14.01, 15.39]	*P* < 0.001, [11.69, 13.34]	*P* < 0.001, [0.28, 0.31]	*P* < 0.001, [0.28, 0.33]	*P* < 0.001, [−5.10, −4.61]	*P* < 0.001, [−10.55, −10.03]
	FedFDD	46.4869 ± 2.5178	38.6840 ± 2.1607	0.9795 ± 0.0030	0.9325 ± 0.0367	1.6851 ± 0.7619	1.4478 ± 1.1673
		*P* < 0.001, [15.30, 16.30]	*P* < 0.001, [11.91, 12.95]	*P* < 0.001, [0.28, 0.31]	*P* < 0.001, [0.28, 0.32]	*P* < 0.001, [−5.31, −4.79]	*P* < 0.001, [−10.99, −10.35]
	FedM2CT	46.7953 ± 2.7223	40.6830 ± 3.0206	0.9803 ± 0.0022	0.9463 ± 0.0119	1.4769 ± 0.3541	1.1180 ± 0.4923
		*P* < 0.001, [15.68, 16.75]	*P* < 0.001, [14.30, 15.33]	*P* < 0.001, [0.28, 0.31]	*P* < 0.001, [0.30, 0.34]	*P* < 0.001, [−5.40, −4.97]	*P* < 0.001, [−11.19, −10.75]
2nd client	FBP	27.6894 ± 2.6477	15.8029 ± 2.4141	0.6324 ± 0.1149	0.3715 ± 0.1617	10.4938 ± 3.7203	56.6852 ± 6.1354
	iRadonMAP	36.0205 ± 2.6284	25.6515 ± 2.6813	0.9570 ± 0.0176	0.7771 ± 0.0168	2.4937 ± 1.8247	19.0679 ± 1.6525
		*P* < 0.001, [7.71, 9.12]	*P* < 0.001, [8.81, 10.27]	*P* < 0.001, [0.29, 0.34]	*P* < 0.001, [0.36, 0.42]	*P* < 0.001, [−9.29, −7.71]	*P* < 0.001, [−38.16, −35.63]
	FedAvg	35.9824 ± 2.5629	16.5101 ± 2.6547	0.9406 ± 0.0186	0.7004 ± 0.0160	3.6761 ± 1.7225	29.5104 ± 1.7596
		*P* < 0.001, [7.61, 8.90]	*P* = 0.05, [−0.05, 1.26]	*P* < 0.001, [0.27, 0.32]	*P* < 0.001, [0.29, 0.35]	*P* < 0.001, [−7.72, −6.19]	*P* < 0.001, [−28.09, −25.34]
	FedProx	36.1136 ± 2.5801	15.9225 ± 2.6638	0.9428 ± 0.0173	0.7225 ± 0.0145	3.9654 ± 1.8992	27.6182 ± 1.8063
		*P* < 0.001, [7.57, 8.99]	*P* = 0.63, [−0.79, 0.63]	*P* < 0.001, [0.27, 0.32]	*P* < 0.001, [0.31, 0.37]	*P* < 0.001, [−7.29, −5.72]	*P* < 0.001, [−29.57, −27.07]
	FedBN	37.4142 ± 2.4206	18.9452 ± 2.5935	0.9511 ± 0.0206	0.7246 ± 0.0166	3.0769 ± 1.3492	28.9762 ± 1.3396
		*P* < 0.001, [9.52, 10.87]	*P* < 0.001, [2.21, 3.64]	*P* < 0.001, [0.28, 0.33]	*P* < 0.001, [0.31, 0.37]	*P* < 0.001, [−8.37, −6.72]	*P* < 0.001, [−28.34, −25.73]
	HyperFed	36.9653 ± 2.4863	22.9323 ± 2.6506	0.9613 ± 0.0187	0.7664 ± 0.0163	3.0203 ± 1.3648	21.5367 ± 1.3689
		*P* < 0.001, [8.64, 10.28]	*P* < 0.001, [6.62, 7.89]	*P* < 0.001, [0.29, 0.34]	*P* < 0.001, [0.35, 0.41]	*P* < 0.001, [−8.24, −6.84]	*P* < 0.001, [−35.77, −33.30]
	FedFDD	37.7009 ± 2.3010	23.0315 ± 2.6614	0.9690 ± 0.0183	0.8027 ± 0.0277	2.6859 ± 1.4635	11.6066 ± 3.1797
		*P* < 0.001, [9.22, 10.65]	*P* < 0.001, [6.42, 7.80]	*P* < 0.001, [0.30, 0.35]	*P* < 0.001, [0.39, 0.45]	*P* < 0.001, [−8.99, −7.42]	*P* < 0.001, [−46.54, −43.67]
	FedM2CT	38.4713 ± 2.2285	28.2812 ± 2.3832	0.9878 ± 0.0211	0.8205 ± 0.0178	1.6468 ± 1.0538	9.4236 ± 1.3842
		*P* < 0.001, [10.31, 11.82]	*P* < 0.001, [11.82, 13.13]	*P* < 0.001, [0.32, 0.36]	*P* < 0.001, [0.40, 0.47]	*P* < 0.001, [−9.83, −8.31]	*P* < 0.001, [−48.15, −45.53]
3rd client	FBP	28.9401 ± 2.7952	18.6741 ± 1.5316	0.6047 ± 0.0821	0.5052 ± 0.0654	9.5862 ± 4.3658	31.3735 ± 6.0536
	iRadonMAP	35.4722 ± 4.5928	28.1728 ± 5.2607	0.9464 ± 0.0264	0.7283 ± 0.0279	3.5861 ± 3.4078	12.2269 ± 3.7998
		*P* < 0.001, [5.42, 7.52]	*P* < 0.001, [8.21, 10.31]	*P* < 0.001, [0.34, 0.37]	*P* < 0.001, [0.21, 0.23]	*P* < 0.001, [−7.18, −5.13]	*P* < 0.001, [−21.38, −18.90]
	FedAvg	34.8057 ± 3.9552	27.5486 ± 4.7072	0.8973 ± 0.0228	0.6534 ± 0.0221	7.4448 ± 3.0777	19.3498 ± 3.8595
		*P* < 0.001, [4.92, 6.73]	*P* < 0.001, [8.13, 9.85]	*P* < 0.001, [0.29, 0.32]	*P* < 0.001, [0.14, 0.16]	*P* < 0.001, [−2.96, −1.12]	*P* < 0.001, [−14.28, −11.59]
	FedProx	34.5706 ± 4.4223	28.5361 ± 4.7724	0.9069 ± 0.0379	0.7004 ± 0.0366	6.4251 ± 4.3185	15.6165 ± 4.4564
		*P* < 0.001, [4.08, 6.33]	*P* < 0.001, [8.35, 10.32]	*P* < 0.001, [0.30, 0.33]	*P* < 0.001, [0.19, 0.22]	*P* < 0.001, [−4.84, −2.25]	*P* < 0.001, [−17.96, −14.69]
	FedBN	36.1691 ± 3.6047	29.2211 ± 4.0491	0.9234 ± 0.0262	0.7916 ± 0.0218	5.3734 ± 3.4713	11.4921 ± 2.8606
		*P* < 0.001, [6.09, 7.49]	*P* < 0.001, [10.05, 11.91]	*P* < 0.001, [0.31, 0.34]	*P* < 0.001, [0.27, 0.30]	*P* < 0.001, [−5.16, −3.19]	*P* < 0.001, [−21.69, −19.09]
	HyperFed	37.4747 ± 3.9946	28.1095 ± 5.4243	0.9402 ± 0.0186	0.6014 ± 0.0463	5.4973 ± 3.2581	24.1474 ± 3.7321
		*P* < 0.001, [7.23, 9.35]	*P* < 0.001, [8.03, 10.32]	*P* < 0.001, [0.33, 0.36]	*P* < 0.001, [0.09, 0.12]	*P* < 0.001, [−5.29, −3.21]	*P* < 0.001, [−9.71, −6.86]
	FedFDD	37.5419 ± 2.3423	34.0508 ± 3.3862	0.9496 ± 0.0152	0.8683 ± 0.0368	3.9496 ± 2.2534	10.9468 ± 4.1693
		*P* < 0.001, [7.53, 8.94]	*P* < 0.001, [15.18, 16.54]	*P* < 0.001, [0.34, 0.37]	*P* < 0.001, [0.35, 0.38]	*P* < 0.001, [−6.43, −4.59]	*P* < 0.001, [−22.51, −19.47]
	FedM2CT	38.8917 ± 3.6181	34.4814 ± 4.7106	0.9619 ± 0.0217	0.8883 ± 0.0294	3.1526 ± 2.9523	8.6756 ± 3.5352
		*P* < 0.001, [9.10, 10.90]	*P* < 0.001, [14.46, 16.63]	*P* < 0.001, [0.35, 0.38]	*P* < 0.001, [0.37, 0.40]	*P* < 0.001, [−7.71, −5.63]	*P* < 0.001, [−24.59, −21.89]

### CT reconstruction performance in situation 2

**Fig. 6.**
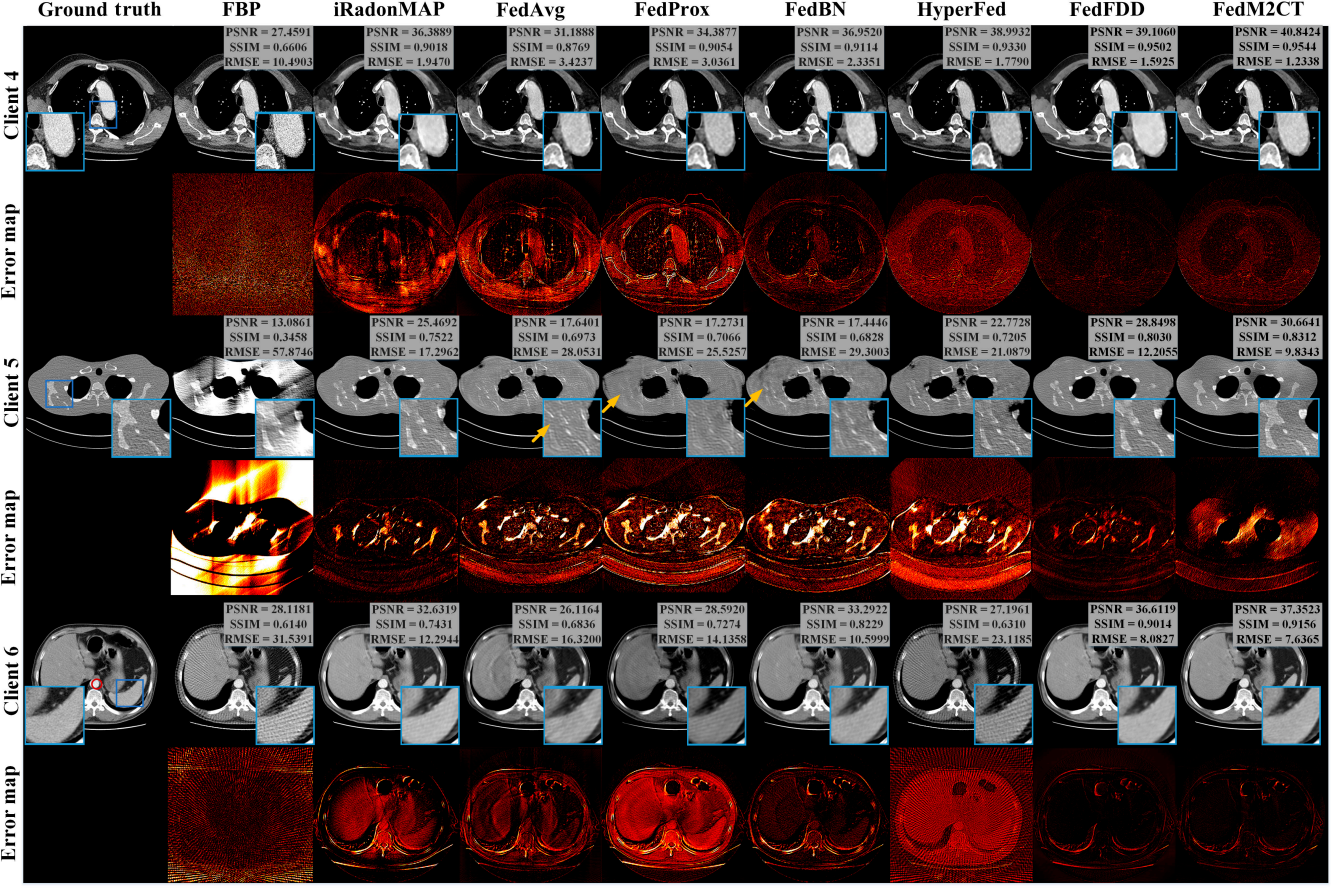
Visual comparisons of the representative images reconstructed by different methods from 3 clients in situation 2. Zoomed-in ROIs indicated by the red boxes are shown on the corresponding images. The quantitative measurements are also listed. The display windows of client 4 and client 6 are [−160,240] HU for the CT images and the zoomed-in ROIs, and the display windows of client 5 are [−400,400] HU for the CT images and the zoomed-in ROIs.

Fig. 6 shows the qualitative comparison between FedM2CT and the other competing methods for 3 patient examples from 3 different clients in situation 2, representing 3 different low-dose sampling conditions. The error maps are calculated as the difference between the reconstructed images and the ground truth. As we can see, the original low-dose CT images suffer from not only high noise levels but also highly biased quantification errors due to insufficient sampling. iRadonMAP, trained with local datasets, can suppress most noise-induced artifacts in all 3 different cases but still has some residual artifacts in the final images at client 6. At client 6, FedAvg, FedProx, FedBN, and HyperFed can reconstruct CT images with varying degrees of noise suppression. However, they still fail to accurately recover structural details, producing undesired artifacts at client 5 and client 6, with relatively high errors found in the skeletal region as indicated by the yellow arrows. For example, at client 5, FedAvg, FedProx, and FedBN tend to smooth edges around the zoomed-in skeletal regions indicated by the yellow arrows. At client 6, HyperFed fails to remove streak artifacts. FedFDD at client 4 and client 6 can provide images with better denoising and artifact suppression than previous FL methods, but the reconstructed images at client 5 fail to recover the anatomical structures, particularly at the bone junctions. In the rightmost column, FedM2CT leverages metadata on the server to learn structured features for global potential alignment, aiding in the reconstruction of fine features across all 3 cases via the CPML module. Thus, FedM2CT has the best reconstruction performance in terms of removing noise-induced artifacts and preserving details across 3 different low-dose sampling conditions, as compared to ground truth. Moreover, FedM2CT improves the PSNR score from 39.1060 to 40.8424 at client 4, from 28.8498 to 30.6441 at client 5, and from 36.6119 to 37.3523 at client 6, surpassing the second-best methods.

**Fig. 7.**
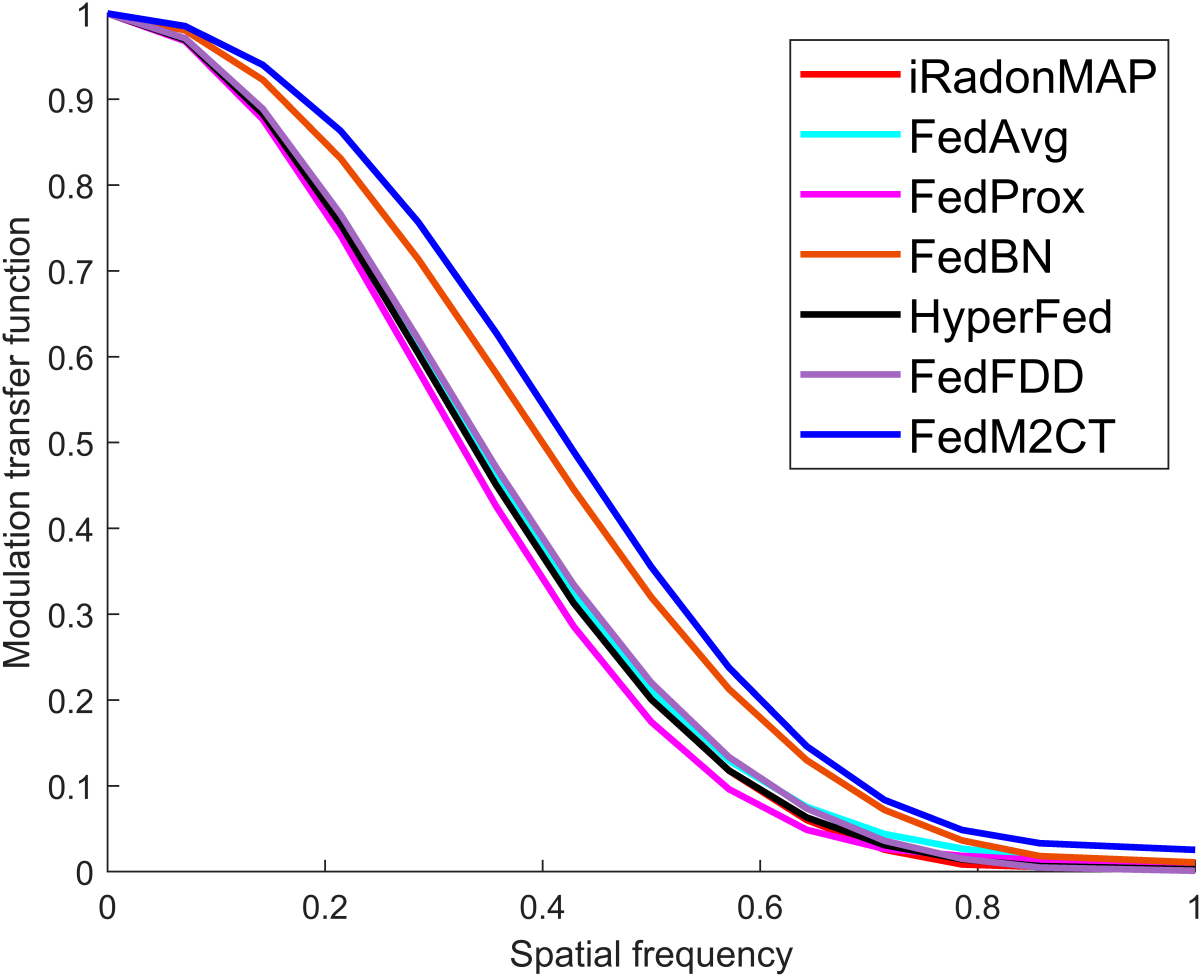
Modulation transfer function (MTF) curves for the desired ROIs indicated by the red circle at client 6 in situation 2 in Fig. 6.

Fig. 7 shows the modulation transfer function curves for the desired ROIs indicated by the red circle at client 6 in Fig. 6. The modulation transfer function curves demonstrate that FedM2CT exhibits higher values compared to the other competing methods, indicating that FedM2CT achieves the best spatial resolution. Table 3 summarizes the average PSNR, SSIM, and RMSE scores with standard deviations in situation 2 and also reports *P* values and 95% CIs derived from the Wilcoxon signed-rank test for each method compared with FBP. These objective metrics confirm that FedM2CT produces significantly higher PSNR and SSIM values and a significantly lower RMSE value than FBP (*P* < 0.001 for all 3 metrics). Moreover, the CIs demonstrate that FedM2CT’s improvements over FBP are statistically significant and exceed those of other FL-based methods, underscoring its superior reconstruction performance.

### CT reconstruction performance in situation 3

**Fig. 8.**
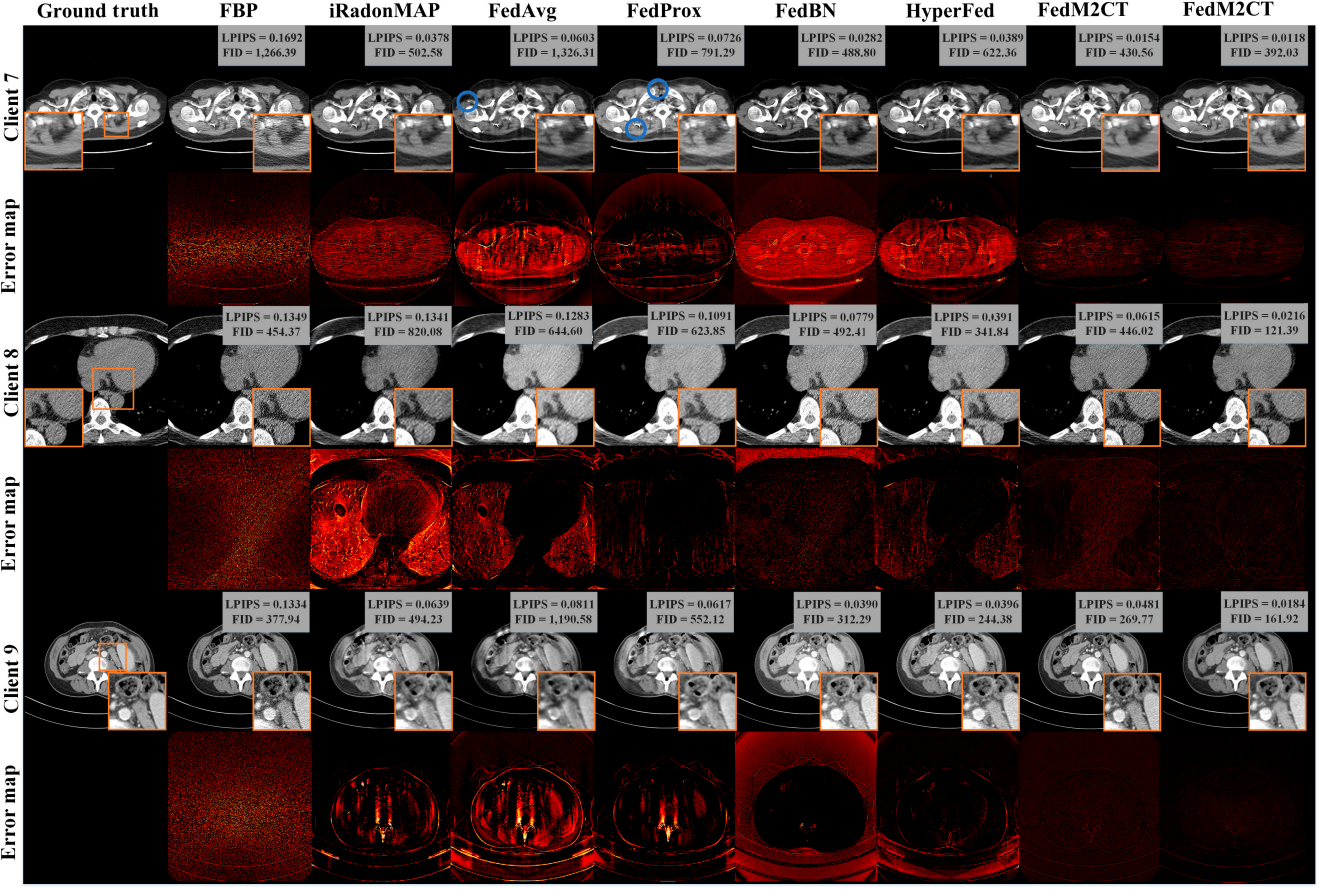
Visual comparisons of the representative images reconstructed by different methods from 3 clients in situation 3. Zoomed-in ROIs indicated by the red boxes are shown on the corresponding images. The quantitative measurements are also listed. The display windows are [−160, 240] HU for the CT images and the zoomed-in ROIs. LPIPS, learned perceptual image patch similarity; FID, Fréchet inception distance.

Fig. 8 shows the qualitative comparison among all of the competing methods in situation 3, where client 7 is treated as supervised learning, while both client 8 and client 9 are treated as unsupervised learning. Compared to the other competing methods, FedM2CT not only achieves a higher FID and a lower LPIPS quantitatively but also shows a smaller error in the zoomed-in ROIs, as evidenced by the error map. For example, at client 7, both FedAvg and FedProx produce undesired artifacts in the final images as indicated by blue circles. In the unsupervised learning cases, it can be seen that the previous FL-based methods fail to suppress the noise-induced artifacts effectively at client 8 and tend to produce oversmoothed results, leading to degraded performance. In this situation, FedFDD is constrained by the differing supervision across clients: It can provide noise-suppressed reconstructions for the supervised client 7 but struggles to effectively suppress noise in the unsupervised clients 8 and 9. This limitation arises because FedFDD depends on paired data to accurately learn the frequency-domain decomposition, which is unavailable in the unsupervised setting, thereby hindering its ability to distinguish between anatomical structures and noise. In contrast, FedM2CT consistently reconstructs detailed, high-quality CT images. The primary reason is that FedM2CT leverages the prior latent features on the server’s metadata, enabling it to recover structure details more efficiently than those without such metadata on the server. Moreover, benefiting from the mutual knowledge-sharing mechanism, CPML enables local models to indirectly learn global features, thereby mitigating the heterogeneous impact of aggregated model.

Three radiologists were invited to independently evaluate the image quality of the reconstruction results from various competing methods. The images were anonymized to ensure unbiased assessment by the radiologists. The radiologists rated image quality on a 5-point scale, considering factors such as denoising level, clarity, artifact severity, structural fidelity, and overall image quality. Table 4 lists the corresponding subjective scores for the reconstruction results. FedM2CT exhibits the highest scores among all cases, indicating its superiority in high-fidelity CT reconstruction. To quantify the inter-rater consistency, the intraclass correlation coefficient was calculated using a 2-way mixed-effects model with absolute agreement. The resulting intraclass correlation coefficient value was 0.992, indicating an exceptionally high level of agreement among the 3 radiologists.

**Table 4.** Subjective quality scores (mean ± standard deviation) from 3 radiologists on the reconstructions from different methods in situation 3

Client	Ground truth	FBP	iRadonMAP	FedAvg	FedProx	FedBN	HyperFed	FedFDD	FedM2CT
Client 7	5.00 ± 0.00	1.28 ± 0.06	4.00 ± 0.16	2.70 ± 0.16	3.10 ± 0.16	3.50 ± 0.16	4.00 ± 0.25	4.07 ± 0.17	4.13 ± 0.21
Client 8	5.00 ± 0.00	1.12 ± 0.10	2.32 ± 0.18	2.30 ± 0.25	2.20 ± 0.16	4.10 ± 0.16	3.90 ± 0.16	4.00 ± 0.16	4.20 ± 0.16
Client 9	5.00 ± 0.00	1.28 ± 0.14	1.92 ± 0.18	2.10 ± 0.16	2.20 ± 0.16	3.60 ± 0.16	4.00 ± 0.16	4.40 ± 0.16	4.50 ± 0.16

### Computational complexity

**Fig. 9.**
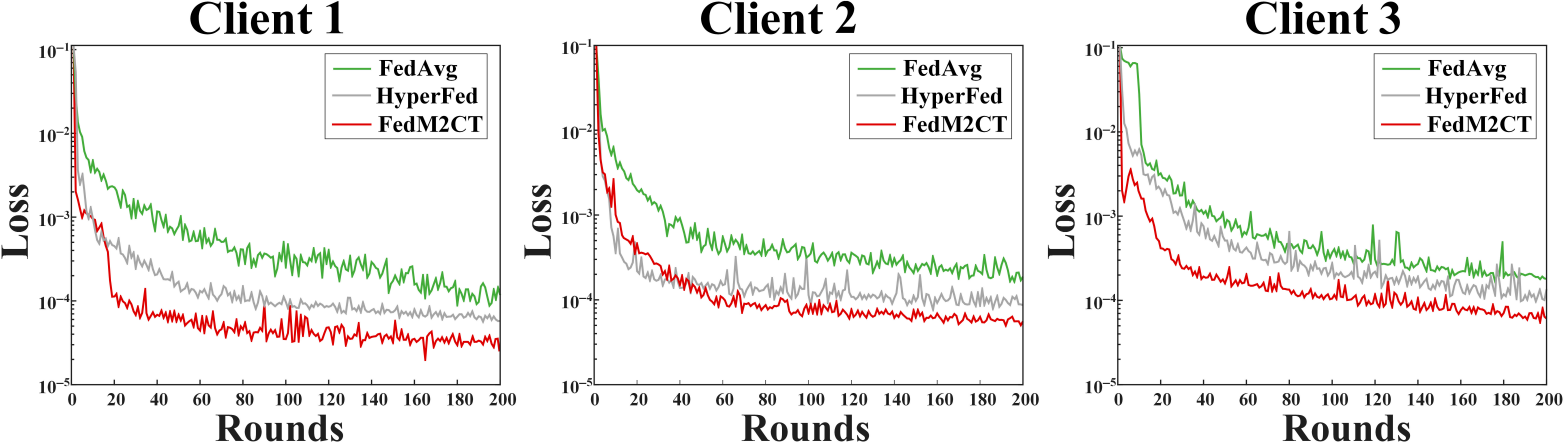
Training curves of FedAvg, HyperFed, and FedM2CT with respect to the number of rounds in situation 1.

In the FL-based methods, the model size at each client, the model size transferred between each client and the server, and local training time are the main concerns. Table 5 summarizes the computational complexity comparison among all of the competing FL-based methods, i.e., the number of local model parameters (*N*_local_), the number of the aggregated components within the model communicated with the server (*N*_comm_), training time per batch (*T*_train_), inference time per batch (*T*_infer_), and video random access memory use (*M*_VRAM_). In the implementation, FedAvg and FedProx completely transfer local client models, while FedBN retains the compact BN layer locally, leaving a portion of model that needs to be communicated. In contrast, HyperFed and FedFDD adopt key component communication. HyperFed uploads the imaging network, while the hyperparameter network is retained locally. FedFDD uploads the high-frequency domain network, while the low-frequency domain network is retained locally. FedM2CT preserves the local client models and uploads only the lightweight shared model in CPML for aggregation, leading to a lower communication load. All methods have comparable training time and inference time. It should be noted that with relatively longer training time and inference time, FedM2CT has memory use comparable to those of the other competing methods. Moreover, Fig. 9 shows the training curves of FedAvg, HyperFed, and the proposed FedM2CT with respect to the number of rounds. With the help of FMDL and CPML, FedM2CT is asymptotically faster than both FedAvg and HyperFed.

**Table 5.** Computational complexity of all FL-based CT reconstruction methods in situation 1

Metric	FedAvg	FedProx	FedBN	HyperFed	FedFDD	FedM2CT
*N*_local_	2.502	2.502	2.502	2.834	4.769	3.261
*N*_comm_	2.502	2.502	2.500	2.502	2.268	0.759
*T*_train_	1.121	1.235	1.122	1.248	1.417	1.303
*T*_infer_	0.844	0.843	0.839	0.932	0.980	0.909
*M*_VRAM_	1.756	1.756	1.756	1.790	1.836	1.776

*N*_local_, number of local model parameters (millions); *N*_comm_, number of the aggregated components within the model communicated with the server (millions); *T*_train_, training time per batch (s); *T*_infer_, inference time per batch (s); *M*_VRAM_, video random access memory use (GB)

### Ablation studies

**Fig. 10.**
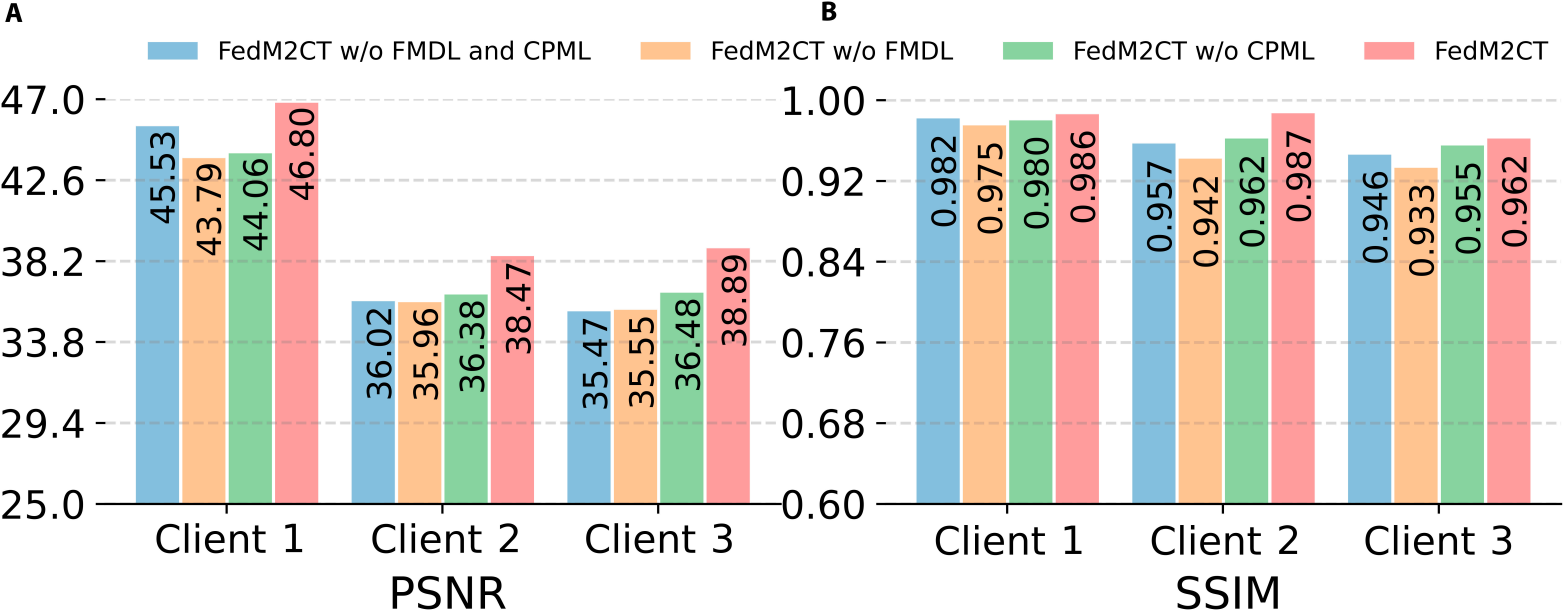
Performance of the proposed FedM2CT and ablated variants in situation 1: (A) PSNR and (B) SSIM results. A variant omitting both FMDL and CPML (FedM2CT without FMDL and CPML), a variant omitting FMDL (FedM2CT without FMDL), and a variant omitting CPML (FedM2CT without CPML).

We conducted ablation experiments to evaluate the effectiveness of FMDL and CPML on the reconstruction performance of FedM2CT. Fig. 10 shows the quantitative assessments (PSNR scores and SSIM scores) of FedM2CT without FMDL and CPML (blue), FedM2CT without FMDL (orange), FedM2CT without CPML (green), and FedM2CT (red) in situation 1. The results indicate that FedM2CT outperforms the other 3 competing methods in both PSNR and SSIM measurements across all cases. This superior performance is primarily due to high-quality paired CT images of FMDL, which can sufficiently provide prior information for task-specific reconstruction at each client. In addition, by mitigating the heterogeneous impact of the aggregated model, the mutual knowledge-sharing strategy in CPML based on the conditional prompt can also help each client achieve all-in-one reconstruction.

We conducted parameter ablation experiments to investigate the impact of critical hyperparameters ($\omega_{c}$, δ, and $\alpha_{k}$) and the conditional prompt (CP) on FedM2CT’s performance. As shown in Table 6, $\omega_{c}$ controls the aggregation weight between server-side metadata and client parameters. The PSNR peaks at $\omega_{c}=0.5$ (38.47 dB), indicating balanced integration of global metadata and local knowledge. Performance declines with a higher $\omega_{c}$, suggesting that overreliance on metadata diminishes client-specific adaptation. For the Huber loss threshold δ, smaller values (e.g., δ=0.1, PSNR = 38.47 dB) outperform larger ones, as they better balance noise suppression and detail preservation in CPML. At =1.0, equivalent to mean squared error loss, performance drops to 37.33 dB, validating Huber loss’s robustness. The mutual learning weight $\alpha_{k}$ achieves optimal results at $\alpha_{k}=0.5$ (38.47 dB), highlighting the importance of harmonizing task-specific reconstruction and mutual knowledge distillation. Disabling CP (PSNR = 36.71 dB) severely degrades performance, while enabling it (PSNR = 38.47 dB) demonstrates its necessity in encoding task-specific sampling conditions. Based on these trends, we selected $\omega_{c}=0.55$, δ=0.1, and $\alpha_{k}=0.5$ to maximize reconstruction fidelity while mitigating data heterogeneity in situation 1.

**Table 6.** Ablation study for the impact of the parameters $\omega_{c}$, δ, $\alpha_{k}$, and CP (the conditional prompt), evaluated using the average PSNR from client 2’s test set in situation 1

Parameter	0.0	0.1	0.2	0.3	0.4	0.5	0.6	0.7	0.8	0.9	1.0
$\omega_{c}$	ND	36.48	35.87	37.29	37.03	38.47	38.15	36.63	36.02	34.33	ND
δ	ND	38.47	38.32	37.99	38.25	38.12	37.84	37.09	37.21	37.65	37.33
$\alpha_{k}$	ND	36.11	ND	37.50	ND	38.47	ND	38.31	ND	37.64	ND
CP	36.71	ND	ND	ND	ND	ND	ND	ND	ND	ND	38.47

### Weight divergence measurement

**Fig. 11.**
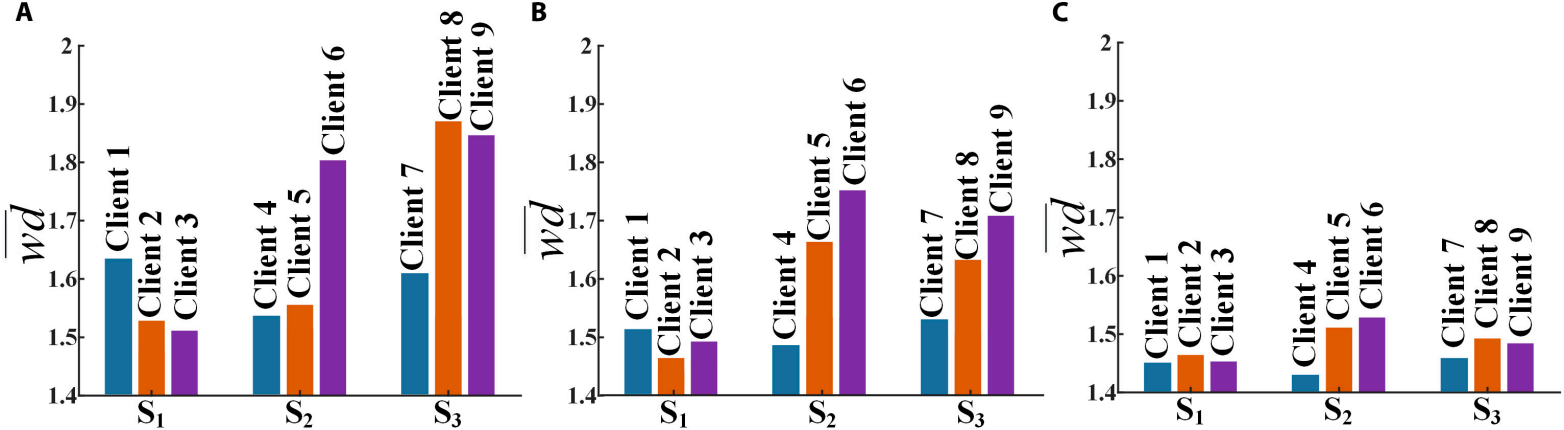
Weight divergence ($\bar{\mathrm{wd}}$) of (A) FedAvg, (B) HyperFed, and (C) the proposed FedM2CT in situations 1 to 3.

As shown in Fig. 1, CT datasets from different sources have a large data heterogeneity due to different CT scanners, different sampling protocols, various subject cohorts, etc. We utilize the weight divergence [18] to characterize the distance between the distribution of each client and server, and it can be used to measure the ability of FL-based methods in mitigating the effect of data heterogeneity among different clients. Smaller weight divergence measurements indicate greater ability in mitigating the effect of the data heterogeneity issue. Weight divergence $\bar{\mathrm{wd}}$ can be defined as follows:

$$\bar{\mathrm{wd}}=\sum_{l=1}^{L}\frac{\left\|\theta_{l}^{\mathrm{FL}}-\theta_{l}^{\text{local}}\right\|}{\left\|\theta_{l}^{\text{local}}\right\|}$$ (10)

where L is the number of layers and $\theta_{l}^{\mathrm{FL}}$ and $\theta_{l}^{\text{local}}$ denote the weight parameters of the FL model and local model, respectively. Fig. 11 shows the weight divergence of FedAvg, HyperFed, and the proposed FedM2CT in situations 1 to 3. It is worth noting that the weight divergence of FedAvg increases as the local data become more heterogeneous, as shown in Fig. 11A. Meanwhile, FedM2CT produces smaller $\bar{\mathrm{wd}}$ values than the other 2 competing methods, indicating that FedM2CT can successfully mitigate the effect of data heterogeneity with promising reconstruction performance.

### Out-of-distribution performance under mixed sampling conditions

**Fig. 12.**
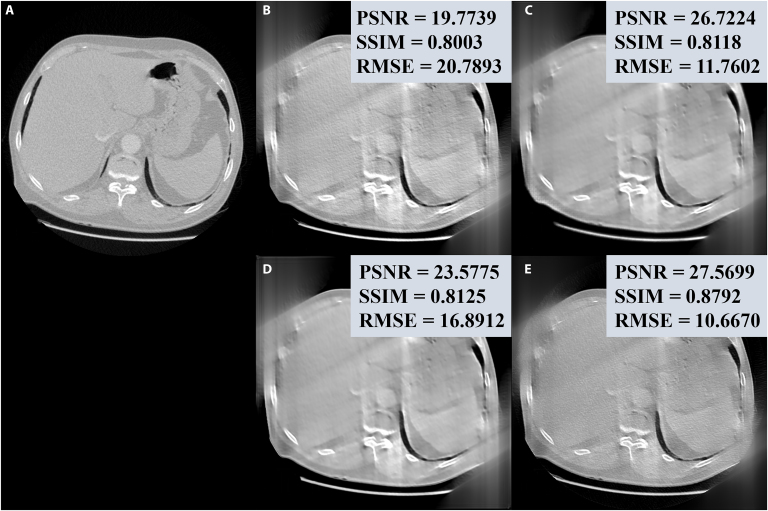
Test results of different methods for limited-angle data with the same geometry from client 4 in situation 2. (A) Ground truth, (B) FBP, (C) FedAvg, (D) FedFDD, (E) FedM2CT. Quantitative metrics, including PSNR, SSIM, and RMSE, are also provided. The display window for all images is set to [−1,000,500] HU.

To comprehensively evaluate the all-in-one capability, we extended the generalization experiments to out-of-distribution situations involving a test set with mixed sampling conditions (low-milliampere-second and limited-angle). Specifically, in situation 2, we simulated limited-angle scanning on the test set of client 4 using its imaging geometry and scan parameters. Therefore, client 4 had the testing data for the mixed sampling conditions. Then, the well-trained models of FedAvg, FedFDD, and FedM2CT for client 4 were tested. As shown in Fig. 12, FBP-reconstructed images exhibit severe anatomical structure loss and blurred textural details. Compared to FedAvg, FedFDD demonstrates inferior generalization performance due to its locally learned low-frequency anatomical features, which fail to recover information missing from limited-angle sampling, resulting in residual artifacts. FedM2CT outperforms competing methods in both reconstruction quality and quantitative metrics. While FedM2CT does not fully restore anatomical structures, the reconstructed images (Figs. 12E) show enhanced reconstruction fidelity. This improvement is attributed to FMDL and CPML, which enable FedM2CT’s task-specific local model to leverage latent global features from metadata while adapting to out-of-distribution data via the conditional prompt.

## Discussion

In this paper, we propose a novel FL method (FedM2CT) for all-in-one CT reconstruction, which consists of 3 modules: TS-iRadonMAP, CPML, and FMDL. Specifically, TS-iRadonMAP can be trained effectively on local private datasets at each client, which consists of a sinogram privacy-preserving submodule and parameter mutual sharing submodule. Each client can maintain a private model with any architecture, and the information in the image will be transmitted to the server by CPML. The CPML module employs mutual learning for bidirectional knowledge distillation, enabling efficient information exchange between clients and servers. The FMDL module leverages high-quality metadata for additional supervised training, adaptively integrating metamodel parameters with local model parameters to mitigate the effect of data heterogeneity. Moreover, the knowledge of task-specific sampling conditions is incorporated into CPML and FMDL through a conditional parameter vector based on the conditional prompt method, effectively modulating the reconstruction process. Therefore, FedM2CT can learn the critical properties of prior knowledge from high-quality metadata and efficiently reconstruct client-specific CT images across different tasks. The comprehensive experiments on publicly available CT and private clinical datasets validate the effectiveness of FedM2CT in all-in-one reconstruction tasks, including low-milliampere-second CT reconstruction, sparse-view CT reconstruction, limited-angle CT reconstruction, and unsupervised low-dose CT reconstruction. The experimental results in Results show that FedM2CT achieves superior performance in all-in-one CT reconstruction compared with several other competing methods in terms of qualitative and quantitative assessment.

There are several key advantages of the proposed FedM2CT. First of all, unlike the traditional FL framework without metadata on the server, FedM2CT holds high-quality metadata collected from publicly available datasets with diverse imaging geometries and sampling protocols, containing a diversity of critical characteristics. This server-side metadata allow FedM2CT to enhance the reconstruction performance at each client by leveraging this rich source of knowledge. As demonstrated in the “Computational complexity” section, the use of metadata can boost the efficiency of the FL-based framework. Second, since each client usually has multiple imaging geometries and sampling protocols, the network must adapt to inputs with different noise levels. FedM2CT takes the task-specific sampling conditions as additional input, thus enabling sampling-conditions-aware reconstruction. It should be noted that both HyperFed and FedM2CT introduce task-specific sampling conditions into network training, and the results from 3 different situations can demonstrate their ability to personalize CT reconstruction. Third, we introduce the mutual learning strategy into CT imaging model learning for the first time to better adapt to different imaging tasks and mitigate the effect of data heterogeneity. With CPML, the networks at each client remain flexible and can be constructed based on the data characteristics and reconstruction task while maintaining effective cross-client knowledge transfer.

Although FedM2CT can improve imaging performance for all-in-one reconstruction, there are 4 limitations when directly applying FedM2CT to realistic medical scenes. First, FMDL requires collecting diverse CT data on the server. The varied distributions of these data are caused by differences in imaging geometries, sampling protocols, and subject cohorts, which may potentially violate privacy-preserving learning requirements and pose a bottleneck for practical training in real-world healthcare situations. Building a fair and rational metadataset is challenging, and ethical concerns such as patient consent and data ownership must be addressed by integrating differential privacy mechanisms [41] and federated metadata anonymization techniques (e.g., *k*-anonymity and data obfuscation) [42]. Second, this study includes simulation experiments only to demonstrate FedM2CT’s performance, thus making it a retrospective analysis; in future work, we will evaluate the method on a larger cohort of prospectively collected data. Third, while our method has slightly higher local computational costs than traditional FL, these can be mitigated through model compression (e.g., dual-side low-rank approximation) [43] and edge computing strategies for local aggregation [44]. Finally, some parameters in FedM2CT need careful tuning. Parameter selection remains an open challenge for all CT reconstruction tasks. Automated hyperparameter optimization (e.g., Bayesian optimization and population-based training) [45] and domain adaptation techniques such as generative-adversarial-network-based metadata simulation [46] could systematically address this challenge. Some advanced network designs or DL-based models can be adapted, such as meta-learning models [47] and large language models [48]. Specifically, we can use large language models to modulate the intermediate features in FedM2CT, thus enabling personalized CT reconstruction. Deploying these advanced models in our FedM2CT could potentially further improve reconstruction performance, representing an important direction in the future.

The main focus of this study is to build an all-in-one reconstruction model to reconstruct CT images from different tasks simultaneously. Therefore, we employed federated metadata-constrained learning and mutual learning, which offer high performance in medical imaging task in previous studies [24,29]. Additional research is needed to evaluate the contribution of different design elements in the adopted methods on the performance of FedM2CT. Future studies should explore the utility of alternative reconstruction strategies such VVBP-Net [2] and 3pADMM [10], as well as the effect of different parameter weight settings on different reconstruction tasks. Recent studies have shown that a diffusion model may be beneficial especially in low-dose CT image reconstruction tasks [49,50]. Exploring the potential benefits of integrating various diffusion models in FedM2CT remains important future work.

In this work, we propose a federated metadata-constrained iRadonMAP framework with mutual learning for all-in-one CT reconstruction, aimed at effectively reconstructing high-quality CT images for different tasks simultaneously. Specifically, the metadata on the server can guide the client to reconstruct task-specific CT images with high fidelity. Furthermore, mutual learning allows each client to construct flexible networks tailored to specific tasks, unlike the fixed network architectures in the traditional FL framework, thereby improving the quality of task-specific reconstruction at each client. In the future, we will adopt the FedM2CT method to other CT applications, such as helical CT imaging and perfusion CT imaging.

## Data Availability

The code is available at https://github.com/wh888s/FedM2CT.
